# Dissection of the *Ren6* and *Ren7* powdery mildew resistance loci in *Vitis piasezkii* DVIT2027 using phased parental–progeny genomes and intraspecific locus graph reconstruction

**DOI:** 10.1093/g3journal/jkaf250

**Published:** 2025-10-21

**Authors:** Mélanie Massonnet, Rosa Figueroa-Balderas, Noé Cochetel, Summaira Riaz, Dániel Pap, M Andrew Walker, Dario Cantu

**Affiliations:** Department of Viticulture and Enology, University of California Davis, Davis, CA 95616, United States; Department of Viticulture and Enology, University of California Davis, Davis, CA 95616, United States; Department of Viticulture and Enology, University of California Davis, Davis, CA 95616, United States; U.S. Department of Agriculture-Agricultural Research Service, San Joaquin Valley Agricultural Center, Parlier, CA 93648, United States; Department of Viticulture and Enology, University of California Davis, Davis, CA 95616, United States; Department of Viticulture and Enology, University of California Davis, Davis, CA 95616, United States; Department of Viticulture and Enology, University of California Davis, Davis, CA 95616, United States; Genome Center, University of California Davis, Davis, CA 95616, United States

**Keywords:** powdery mildew resistance, *Vitis piasezkii;* NLR genes, Ren6, *Ren7;* haplotype phasing, grapevine breeding

## Abstract

The Chinese grape accession *Vitis piasezkii* DVIT2027 carries 2 loci associated with powdery mildew (PM) resistance, *Ren6* and *Ren7*, which differ in timing and strength of response to *Erysiphe necator*. Both loci are consistent with recognition by intracellular immune receptors. To identify the underlying nucleotide-binding leucine-rich repeat (NLR) genes, we assembled chromosome-scale diploid genomes of DVIT2027 and the susceptible *V. vinifera* F2-35, parents of a segregating F1 population. We integrated these assemblies with deep resequencing data from 8 F1 siblines carrying different *Ren6*/*Ren7* combinations and generated trio-binned, parent-phased genomes for 6 progeny. This resolved both PM-resistant (PMR) and PM-susceptible (PMS) haplotypes at *Ren6* and *Ren7*. Comparative analyses revealed extensive structural variation and complete haplotype specificity among NLRs, with several candidate genes lacking allelic counterparts in PMS haplotypes. Expression profiling across PMR siblines identified 4 and 2 CC-NBS-LRR genes potentially associated with *Ren6* and *Ren7*, respectively. Sequence graph reconstruction of these loci across multiple *V. piasezkii* accessions revealed broad intraspecific diversity and DVIT2027-specific nodes, including within candidate NLR genes. These results provide a high-resolution view of *Ren6* and *Ren7* and support the identification of resistance gene candidates for functional validation and grapevine breeding.

## Teaser

Grapevine powdery mildew is a major disease, and the Chinese grape *Vitis piasezkii* DVIT2027 carries two resistance loci, *Ren6* and *Ren7*. To identify the genes responsible, Massonnet *et al*. generated complete diploid genomes of resistant and susceptible parents and created phased genomes for their progeny. This allowed the authors to resolve resistant and susceptible haplotypes, revealing extensive structural variation and NLR immune receptor genes unique to the resistant haplotypes. Expression analysis and graph-based comparisons across accessions highlighted strong candidate resistance genes. Their results provide a high-resolution framework to dissect complex resistance loci to accelerate grape breeding for durable disease resistance.

## Introduction

Cultivated grapevines (*V. vinifera* spp. *vinifera*; hereafter *V. vinifera*) are generally susceptible to powdery mildew (PM), a disease caused by the obligate biotrophic ascomycete *Erysiphe necator* Schwein. (syn. *Uncinula necator*). *E. necator* infects all grapevine green tissues, including leaves, shoots, inflorescences, and berries before the onset of ripening (Gadoury et al. 2011), reducing photosynthetic capacity and negatively impacting yield, fruit composition, and wine quality (Calonnec et al. 2004; Stummer et al. 2005). PM control relies heavily on prophylactic fungicide applications, which are costly (Sambucci et al. 2019). An alternative approach is the use of resistant cultivars developed through the introgression of PM resistance loci from wild *Vitis* species and *Muscadinia rotundifolia*. To date, 15 loci associated with PM resistance have been mapped and named *Run* (Resistance to *Uncinula necator*) or *Ren* (Resistance to *E. necator*) (Dry et al. 2019; Karn et al. 2021; Ricciardi et al. 2024). Although introgression has conferred partial or complete resistance to the disease, it often introduces linkage drag from wild donor genomes (Foria et al. 2022). The removal of unwanted wild alleles is hampered by low recombination rates in grape breeding programs and the absence of perfect markers, i.e. markers specific to the resistance (*R*) genes, due to limited knowledge of the genes underlying resistance. To date, only one *R* gene, encoding an intracellular receptor, has been characterized, at the *Run1* locus of *M. rotundifolia* G52 (Feechan et al. 2013). Recent diploid genome assemblies of wild grape accessions (Cantu et al. 2024) have enabled the identification of candidate *R* genes at several loci (Paineau et al. 2024), including *Run1.2a*/*b* and *Run2.2* (Massonnet et al. 2022b). In this study, we leveraged recent genomic advances to identify candidate *R* genes associated with *Ren6* and *Ren7* in *V. piasezkii* DVIT2027.

Four *V. piasezkii* accessions collected during the 1980 Sino-American botanical expedition in the Shennongjia Forestry District, Hubei Province, China (DVIT2026, DVIT2027, DVIT2028, and DVIT2032) were found to be PM-resistant (PMR) (Pap et al. 2016). Among these, only DVIT2027 has been genetically characterized. Using quantitative trait locus (QTL) mapping of the F1 population 11373, derived from a cross between the PM-susceptible (PMS) *V. vinifera* F2-35 and *V. piasezkii* DVIT2027, we previously identified 2 *R* loci: *Ren6* on chromosome 9 and *Ren7* on chromosome 19 (Pap et al. 2016). Both loci are heterozygous in DVIT2027 and confer resistance to *Erysiphe necator*, though with distinct response dynamics. *Ren6* provides complete resistance, while *Ren7* results in reduced colony size. In both cases, resistance relies on programmed cell death (PCD) following fungal penetration: at the appressoria of germinated spores for *Ren6* and at the appressoria of secondary hyphae for *Ren7*. As PCD occurs postpenetration, resistance is likely triggered by intracellular recognition of *E. necator* effectors by intracellular receptors encoded within the *Ren6* and *Ren7* loci (Pap et al. 2016).

Nucleotide-binding domain and leucine-rich repeat (NLR) proteins are intracellular receptors that activate plant defense responses upon pathogen recognition (Chia and Carella 2023). Canonical NLRs are composed of 3 domains: a variable N-terminal domain, a central nucleotide-binding site (NBS) domain, and a C-terminal leucine-rich repeat (LRR) domain. The N-terminal domain is typically classified as coiled-coil (CC), Resistance to Powdery Mildew 8 (RPW8), or Toll/interleukin-1 receptor (TIR) type (Lolle et al. 2020). The C-terminal LRR region mediates effector recognition, either directly or indirectly, while the N-terminal domain contributes to the formation of resistosomes, large oligomeric complexes that initiate downstream immune signaling (Chia and Carella 2023; Locci and Parker 2024). NLR activation triggers calcium influx, reactive oxygen species production, and an extensive transcriptional reprogramming (Lolle et al. 2020). It is also commonly associated with the hypersensitive response, a rapid, localized form of PCD at the site of infection (Lolle et al. 2020).

In this study, we aimed to identify NLR genes potentially responsible for PM resistance at *Ren6* and *Ren7* loci of *V. piasezkii* DVIT2027. We first generated diploid, chromosome-scale genome assemblies of *V. piasezkii* DVIT2027 and the maternal parent of the 11373 F1 population, *V. vinifera* F2-35. Since both loci are heterozygous in DVIT2027, we used genomic data from F1 progeny to distinguish *Ren6* and *Ren7* from their corresponding PMS haplotypes and validate their structure and phasing. NLR genes within the 2 *R* loci and their PMS haplotypes were identified, and their gene models were manually curated. To pinpoint NLRs specific to *Ren6* and *Ren7*, we compared each *R* haplotype with its PMS counterpart, focusing on structural variation affecting gene content and domain composition, and examined allelic relationships between NLRs across haplotypes. To prioritize candidate *R* genes, i.e. genes potentially involved in PM resistance, we profiled NLR gene expression in 6 PMR 11373 siblines carrying different combinations of *Ren6* and *Ren7* using RNA sequencing (RNA-seq). Finally, we constructed sequence graphs for the *Ren6* and *Ren7* regions across 4 *V. piasezkii* accessions to assess intraspecific structural diversity and identify graph nodes unique to DVIT2027, including those overlapping candidate NLR genes.

## Materials and methods

### Plant material and PM inoculation

Young leaves from *V. piasezkii* DVIT2027, *V. vinifera* F2-35 (Carignane × Cabernet Sauvignon), and 8 of their progeny (Pap et al. 2016): 11373-158 (*Ren6*^+^/*Ren*7^−^), 11373-195 (*Ren6*^+^/*Ren*7^−^), 11373-137 (*Ren6*^−^/*Ren*7^+^), 11373-257 (*Ren6*^−^/*Ren*7^+^), 11373-098 (*Ren6*^+^/*Ren*7^+^), 11373-145 (*Ren6*^+^/*Ren*7^+^), 11373-130 (*Ren6*^−^/*Ren*7^−^), and 11373-156 (*Ren6*^−^/*Ren*7^−^) were collected for genome sequencing.

For gene expression profiling, PM was inoculated as described in Amrine et al. (2015). Three plants of each grape accession were inoculated with *E. necator* C-train. Meanwhile, 3 other plants were mock-inoculated. At 1 and 5 d postinnoculation (dpi), 2 leaves from each plant were pooled together to constitute a biological replicate and were immediately frozen in liquid nitrogen. Each condition was thus represented by 3 biological replicates.

### DNA and RNA extraction, library preparation, and sequencing

High-molecular-weight genomic DNA was isolated from young leaves of *V. piasezkii* DVIT2027, *V. vinifera* F2-35, and the 8 F1 progeny as in Chin et al. (2016). DNA quantity and purity were assessed using a Qubit™ 1× dsDNA HS Assay Kit (Thermo Fisher, MA, USA) and a Nanodrop 2000 spectrophotometer (Thermo Scientific, IL, USA), respectively. DNA integrity was evaluated with the DNA high-sensitivity kit (Life Technologies, CA, USA) and by pulsed-field gel electrophoresis. Unfortunately, the quantity and integrity of the DNA extracted from the siblines 11373-195 (*Ren6*^+^/*Ren*7^−^) and 11373-137 (*Ren6*^−^/*Ren*7^+^) were not sufficient for preparing long-read DNA sequencing (DNA-seq) libraries. Accordingly, DNA extracted from these 2 samples were only used for short-read DNA-seq. HiFi libraries of *V. piasezkii* DVIT2027 and *V. vinifera* F2-35 were prepared using the SMRTbell Express Template Prep Kit 3.0 (Pacific Biosciences, CA, USA) following the manufacturer's protocol. Libraries were size-selected using a LightBench instrument (Yourgene Health, FL, USA) with a cut-off size range of 10 kbp and then sequenced with a PacBio Revio platform (DNA Technology Core Facility, University of California, Davis, CA, USA). SMRTbell libraries of the 11373 siblines were made following the manufacturer's protocol for the SMRTbell Express Template Prep Kit 2.0 (Pacific Biosciences, CA, USA). Size selection of the libraries was performed using a BluePippin instrument (Sage Science, Beverly, MA, USA) with a cut-off size range of 18 to 80 kbp. After size selection, libraries were sequenced using PacBio Sequel II system (DNA Technology Core Facility, University of California, Davis, CA, USA) (Supplementary Table 1).

DNA extraction and short-read DNA-seq library preparation were performed as Massonnet et al. (2020). DNA libraries were sequenced as 150-bp paired-end reads using an Illumina HiSeq3000 for *V. vinifera* F2-35 library and an HiSeqX Ten system for the others (IDseq, Davis, CA, USA). Total RNA was extracted with a cetyltrimethylammonium bromide (CTAB)-based protocol as described in Cochetel et al. (2021). RNA purity was evaluated with a Nanodrop 2000 spectrophotometer (Thermo Scientific, IL, USA), RNA quantity with a Qubit 2.0 Fluorometer and a broad-range RNA kit (Life Technologies, CA, USA), and RNA integrity through electrophoresis and with an Agilent 2100 Bioanalyzer (Agilent Technologies, CA, USA). cDNA library preparation was made as in Amrine et al. (2015) using the Illumina TruSeq RNA Sample Preparation Kit v2. cDNA library of young leaves of *V. piasezkii* DVIT2027 was sequenced as 100-bp single-end reads with an Illumina HiSeq3000. Concerning the PM- and mock-inoculated leaves of the 11373 siblines, cDNA libraries were sequenced using an Illumina HiSeq2500 sequencer (DNA Technologies Core, University of California, Davis, CA, USA) as 50-bp single-end reads (Supplementary Table 2). Full-length cDNA sequencing (Iso-Seq) library was prepared from young leaves of *V. piasezkii* DVIT2027 and sequenced as in Cochetel et al. (2023). A total of 282,342 Iso-Seq full-length reads with a N50 of 2.51 kbp were obtained (658.15 Mbp). A summary of the DNA and RNA sequencing data generated in this study can be retrieved in Supplementary Table 3.

### Genome assembly

Long-read DNA sequencing of the PacBio HiFi reads of *V. piasezkii* DVIT2027 and *V. vinifera* F2-35 resulted in 139 and 176× of coverage of a 500-Mbp haploid genome, respectively (Supplementary Table 1). PacBio HiFi reads were first subsampled to 60× coverage using seqtk sample from the package seqtk v.1.2-r101-dirty (https://github.com/lh3/seqtk) and the parameter “-s100”, and then assembled using hifiasm (Cheng et al. 2021) with the option “-n 13” (Supplementary Table 4). Contigs were scaffolded into diploid chromosome-scale pseudomolecules using HaploSync v1.0 (Minio et al. 2022) and the consensus grape synteny map. Telomere repeat units were searched as described in Shi et al. (2023), using TIDK v.0.2.63 (https://github.com/tolkit/telomeric-identifier); tidk explore with the options “--minimum 5 --maximum 12 -t 2 --log”; tidk search with the option “-s TTTAGGG”. Results were visualized with tidk plot. BUSCO v.5.4.7 with the library embryophyte_odb10 (Waterhouse et al. 2018) were used to evaluate the gene space completeness of the genome assemblies. Concerning the 6 F1 individuals (11373-158, 11373-137, 11373-098, 11373-145, 11373-130, 11373-156), genome assembly of the PacBio CLR reads was performed with Canu v2.2 and the trio-binning approach with short DNA-seq reads from the 2 parents (Koren et al. 2018), generating 2 haploid, parental-phased assemblies per F1 line.

Canu was performed with the following parameters: genomeSize = 500 m useGrid = false maxMemory = 80 maxThreads = 40. For sequence polishing, PacBio CLR reads were first aligned on the genome assembly using the bioconda package pbmm2 v.1.9.0 (https://github.com/PacificBiosciences/pbmm2). Polishing was then performed with the bioconda package pbgcpp v.2.0.2-2.0.2 (https://github.com/PacificBiosciences/gcpp; downloaded August 11th, 2022) using the Arrow algorithm (Supplementary Table 4).

### Genome annotation

Repetitive elements were annotated using RepeatMasker v.4.1.7-p1 (http://www.repeatmasker.org) and a grape repeat library (Minio et al. 2019). For the gene annotation of *V. piasezkii* DVIT2027, we used as transcriptomic evidence the PN40024 V5.1 predicted protein-coding sequences (https://grapedia.org/t2t_annotation/) and the high-quality transcripts obtained from the Iso-Seq full-length reads of *V. piasezkii* DVIT2027 young leaves. Iso-Seq full-length reads were clustered using IsoSeq v.3 (https://github.com/Pacif​icBiosciences/IsoSeq), leading to 28,807 high-quality transcripts, 194 low-quality transcripts, and 90,709 singletons. For *V. vinifera* F2-35, transcript evidences consisted of the PN40024 V5.1 predicted protein-coding sequences and the high-quality isoforms of Cabernet Sauvignon (Minio et al. 2019). A set of high-quality gene models was created using the transcriptomic evidence aforementioned, the PASApipeline v2.5.3 (https://github.com/PASApipeline/PASApipeline), GMAP v.2024-11-20 (Wu and Watanabe 2005), and pblat v.2.5.1 (Wang and Kong 2019), and was used as input for the *ab initio* predictors Augustus v.3.5.0 (Stanke et al. 2006) and GeneMark v.3.68_lic (Lomsadze et al. 2005). EvidenceModeler v.2.1.0 (Haas et al. 2008) was then used to generate consensus models from the gene models produced by the ab initio predictions and PASA. A total of 75,207 gene models (78,717 transcripts) were found for *V. piasezkii* DVIT2027 and 63,686 gene models (68,243 transcripts) for *V. vinifera* F2-35. Gene models encoding protein sequences with a length less than 50 amino acids, or in-frame stop codons, or no start methionine, or no stop codon, were removed. Therefore, 70,232 transcripts (66,931 gene loci; 2,649 loci with ≥ 2 transcripts) and 64,022 transcripts (56,613 gene loci; 3,922 loci with ≥ 2 transcripts) remained for *V. piasezkii* DVIT2027 and *V. vinifera* F2-35, respectively.

### Functional annotation

Predicted proteins of *V. piasezkii* DVIT2027 and *V. vinifera* F2-35 genomes were aligned onto the proteins of *Arabidopsis thaliana* (Araport11_genes.201606.pep.fasta; https://www.arabidopsis.org/download/index.jsp) using BLASTP v.2.12.0+ (Altschul et al. 1990). Alignments with an identity greater than 30%, and both reference:query and query:reference coverages between 75 and 125%, were retained. Among the remaining alignments, the alignment with the highest product of identity, query coverage, and reference coverage was selected for each grape protein to determine its homolog in *A. thaliana*. Same methods were used to identify homologous proteins in the grape *V. vinifera* PN40024 genome V1 and V5.1 annotations (Jaillon et al. 2007; Shi et al. 2023).

Protein domains were detected using hmmsearch from HMMER v.3.3.1 (http://hmmer.org/) and the Pfam-A hidden markov models (HMM) database (El-Gebali et al. 2019) (downloaded on March 12th, 2025). Protein domains with an independent *E*-value < 1.0, and at least 50% of the HMM, were selected. CC domains were identified using COILS (Lupas et al. 1991). Functional annotation of the predicted proteins of *V. piasezkii* DVIT2027 and *V. vinifera* F2-35 genomes can be retrieved in Supplementary Tables 5 and 6. LRR motifs among the candidate NLRs were predicted using LRRpredictor v1.0 (Martin et al. 2020). The CC, NBS, and LRR domains identified among the NLRs composing *Ren6* and *Ren7* were used to manually annotate them in the genome of *V. piasezkii* DVIT2027.

### NLR gene annotation

Gene loci potentially encoding NLRs were detected among *V. piasezkii* DVIT2027 and *V. vinifera* F2-35 genomes with NLR-Annotator using default parameters (Steuernagel et al. 2020). To assess the intron–exon structure of the potential NLR genes within the *R* loci and the PMS alternative haplotypes, RNA-seq reads from *V. piasezkii* DVIT2027, *V. vinifera* F2-35, and their 8 progeny lines were aligned on the combined genomes of *V. piasezkii* DVIT2027, *V. vinifera* F2-35, and *E. necator* C-strain (Jones et al. 2014), using HISAT2 v.2.1.0 (Kim et al. 2015), and the parameters “--end-to-end --sensitive -k 50”. RNA-seq reads of *V. vinifera* F2-35 were retrieved from the NCBI BioProject PRJNA897013 (Massonnet et al. 2022a). Alignments were visualized in Integrative Genomics Viewer (IGV) v.2.4.14 (Robinson et al. 2011). Manual refinement of the NLR gene models was performed when the alignments of the RNA-seq reads indicated a different intron–exon structure than the ab initio structural annotation.

### *Ren6* and *Ren7* region localization

The loci *Ren6* and *Ren7* and their alternative haplotypes were located by *in silico* amplification of the *Ren6*- and *Ren7*-associated markers (Supplementary Table 7) described in Pap et al. (2016) on the genome assemblies using dipr (https://github.com/douglasgscofield/dispr).

### Haplotype coverage analysis

To distinguish *Ren6* and *Ren7* haplotypes from their PMS alternative haplotypes and confirm their phasing in the genomes of *V. piasezkii* DVIT2027 and *V. vinifera* F2-35, we used the short DNA-seq reads from the 8 F1 lines, *V. piasezkii* DVIT2027 and *V. vinifera* F2-35. Adapter sequences and low-quality reads were removed using Trimmomatic v.0.36 (Bolger et al. 2014) with the following parameters: “LEADING:3 TRAILING:3 SLIDINGWINDOW:10:20 MINLEN:36 CROP:150”. High-quality reads were then aligned onto the combined genomes of *V. piasezkii* DVIT2027 and *V. vinifera* F2-35 using BWA v0.7.17 (Li and Durbin 2009) with default parameters. Reads aligning perfectly (i.e. with no mismatches) to the reference genome were selected with bamtools filter v.2.5.1 (Barnett et al. 2011) and the option “NM:0”, ensuring that only unambiguous alignments were retained. This strict criterion was applied to prevent reads carrying parental SNPs from mapping to both haplotypes, allowing accurate discrimination of parental haplotype blocks and reliable estimation of haplotype-specific coverage. Base coverage was performed using genomecov from BEDTools v2.29.1 (Quinlan 2014). Coverage in repetitive elements was removed using BEDTools intersect. Median coverage per window of 10-kbp was computed by BEDTools map. For each grape accession, the median coverage per 10-kbp window was normalized by dividing by the sequencing coverage of the accession (Supplementary Table 2). Phasing and structure of the *Ren6* and *Ren7* haplotypes and their alternative haplotypes in the genome of *V. piasezkii* DVIT2027 were also confirmed using the alignment of the PacBio CLR reads of 6 of the 8 siblines on the 4 haplotypes. Reads were first subsampled to 40× coverage using seqtk sample from the package seqtk v.1.2-r101-dirty (https://github.com/lh3/seqtk) and the parameter “-s100”, and then aligned onto the combined genomes of *V. piasezkii* DVIT2027 and *V. vinifera* F2-35 with Minimap2 v.2.24-r1122 (Li 2018). Alignments were visualized using IGV v.2.4.14 (Robinson et al. 2011).

### Sequence comparison

Pairwise sequence alignments of whole haplotypes were done with NUCmer from MUMmer v.4.0.0 (Marçais et al. 2018) with the “--mum” option. For the alignment of 11373 siblines’ genomes onto *Ren6*, *Ren7*, and their alternative haplotypes, each parental genome was aligned onto the parental haplotypes. Alignments longer than 5 kbp were retained using the parameters “-l 5000”. Regarding the comparison between PMR and PMS haplotypes, alignments with at least 90% identity are represented in Fig. 2. Structural variants (SVs; > 50 bp) were called using show-diff from MUMmer v.4.0.0 with default parameters. SNPs and INDELs (< 50 bp) were detected using show-snps from MUMmer v.4.0.0. SV coordinates were intersected with the CDS annotation of the NLRs composing *Ren6* and *Ren7* using BEDTools intersect v2.29.1 (Quinlan 2014) to determine the impact of the SVs on the NLR genes. SVs affecting the NLR gene content of *Ren6* and *Ren7* were manually inspected using the alignment results. The potential effect of the SNPs, detected between *Ren6* and *Ren7* and their corresponding PMS alternative haplotype in DVIT2027, on the amino acid content was predicted using SnpEff v.4.3t (Cingolani et al. 2012).

To assess whether NLR genes of *Ren6* and *Ren7* loci were present in *V. piasezkii* 0957, BL-1 and BS-40 (Zhao et al. 2025; Guo et al. 2025a, 2025b), CDS of the NLR genes composing the 2 *R* loci were first aligned onto the 5 haploid genomes using GMAP v.2020-06-01 (Wu and Watanabe 2005) (Supplementary Tables 8 to 10). Resulting alignments were then used to generate proteins by gffread from Cufflinks v.2.2.1 (Trapnell et al. 2010). Sequences of the latter ones were compared to the protein sequences of the NLRs composing *Ren6* and *Ren7* using BLAST v.2.2.28+ (Altschul et al. 1990).

### Phylogenetic analysis

Predicted NLR protein sequences from *Ren6* and *Ren7*, and their PMS alternative haplotypes in *V. piasezkii* DVIT2027 and *V. vinifera* F2-35 were aligned using MUSCLE (Edgar 2004) in MEGAX (Kumar et al. 2018). Phylogenetic analyses were done with MEGAX (Kumar et al. 2018) using the Neighbor-Joining method (Saitou and Nei 1987) and 1,000 bootstrap replicates. Evolutionary distances were computed with the Poisson correction method (Zuckerkandl and Pauling 1965), eliminating the positions with less than 5% site coverage.

### Gene expression analysis

High-quality reads were obtained using Trimmomatic v.0.36 (Bolger et al. 2014) with the following parameters: “LEADING:3 TRAILING:3 SLIDINGWINDOW:10:20 MINLEN:36” (Supplementary Table 11). Transcript abundance was evaluated with Salmon v.1.5.1 (Patro et al. 2017). First, the transcriptome index file was built using the combined protein-coding sequences of *V. piasezkii* DVIT2027 and *V. vinifera* F2-35, and *E. necator* C-strain (Jones et al. 2014), their genomes as decoys, and the options: “-k 13 --keepDuplicates”. Second, transcript abundance was quantified with the parameters “--gcBias --seqBias --validateMappings”. Read counts from *E. necator* C-strain were removed, and gene expression in transcript per million (TPM) from the grape CDS were calculated. Differential gene expression analysis was performed using DESeq2 v.1.42.1 (Love et al. 2014) after importing the quantification files with the R package tximport v.1.30.0 (Soneson et al. 2016).

### Sequence graph construction

Sequence graphs of *Ren6* and *Ren7* and their respective 6 alternative haplotypes were built using the nf-core/pangenome pipeline (Heumos et al. 2024) with NextFlow v.25.04.6 (Di Tommaso et al. 2017) and the parameters “-r 1.1.2 -profile singularity”.

## Results

### Haplotype-resolved genome assemblies of *V. piasezkii* DVIT2027 and *V. vinifera* F2-35

To identify *R* genes potentially involved in PM resistance of *V. piasezkii* DVIT2027, we sequenced the genomes of the paternal (*V. piasezkii* DVIT2027) and maternal (*V. vinifera* F2-35) parents of the 11373 F1 population (Pap et al. 2016), using long-read DNA sequencing (PacBio HiFi reads; Supplementary Table 1). Draft haplotype-resolved genome assemblies comprised 120 contigs for *V. piasezkii* DVIT2027 and 238 contigs for *V. vinifera* F2-35, totaling 1,018.8 and 975.7 Mbp, respectively (Supplementary Table 4). Pseudomolecule reconstruction yielded 2 sets of 19 chromosomes: 504.6 Mbp (Hap1) and 507.1 Mbp (Hap2) for DVIT2027, and 481.3 Mbp (Hap1) and 481.5 Mbp (Hap2) for F2-35 (Table 1). The difference in length between the 2 genome assemblies (38.1 Mbp) was mostly due to longer chromosomes 12, 13, 16, 17, and 18 in DVIT2027 compared to F2-35 (Supplementary Table 12). This size difference was not only made of repetitive elements (49.2%) but also of protein-coding gene loci (12.3%).

**Table 1.** Statistics of the genome assemblies of *V. piasezkii* DVIT2027 and *V. vinifera* F2-35.

	*V. piasezkii* DVIT2027			*V. vinifera* F2-35		
	Hap 1	Hap 2	Unplaced	Hap 1	Hap 2	Unplaced
Assembly length (Mbp)	504.6	507.1	7.6	481.3	481.5	18.4
Number of sequences	19	19	55	19	19	93
Average length (Mbp)	26.6	26.7	0.1	25.3	25.3	0.2
Telomeres	31/38	33/38	0	34/38	33/38	0
Number of gene loci	34,926	31,353	652	29,820	29,399	394
Number of transcripts	37,544	32,036	652	32,015	31,593	414
Gene content (Mbp)	149.6	134.3	1.7	141.9	138.1	0.9
Repeats (Mbp)	284.1	285.0	5.5	270.0	270.0	15.8
Repeats (%)	56.3	56.3	71.9	56.1	56.1	85.9
BUSCO (%)	99.3	99.5	0.6	99.4	99.1	0.2

Unanchored contigs accounted for only 0.7% (DVIT2027) and 1.9% (F2-35) of the total genome size and were predominantly composed of repetitive sequences (Table 1). BUSCO analysis showed high completeness, with 99.3 ± 0.2% of expected single-copy orthologs identified in each haplotype. On average, 32.8 ± 1.3 telomeres of the 38 possible telomeres were detected per haplotype. Together, these results demonstrate that we generated highly contiguous, haplotype-resolved genome assemblies for both parents of the 11373 population.

### Identification and haplotype phasing of *Ren6* and *Ren7* loci of *V. piasezkii* DVIT2027

The boundaries of the *Ren6* and *Ren7* haplotypes, along with their PMS alternative haplotypes, were defined by *in silico* amplification of the *Ren6*-associated markers PN9-057 and VMC4h4.1, and *Ren7*-associated markers VMC9a2.1 and VVIu09 (Pap et al. 2016) on the diploid genomes of DVIT2027 and F2-35. Because *Ren6* and *Ren7* are heterozygous in DVIT2027, we distinguished the PMR haplotypes from their PMS alternatives and validated haplotype phasing by analyzing normalized median base coverage (per 10 kbp) across both parental genomes using deep sequencing data of 8 siblines of population 11373 (Pap et al. 2016), including 2 lines carrying *Ren6* only (*Ren6*^+^/*Ren7*^−^: 11373-158 and 11373-195), 2 with *Ren7* only (*Ren6*^−^/*Ren7*^+^: 11373-137 and 11373-257), 2 carrying both *R* loci (*Ren6*^+^/*Ren7*^+^: 11373-098 and 11373-145), and 2 lacking both (*Ren6*^−^/*Ren7*^−^: 11373-130 and 11373-156). In *Ren6*^+^ accessions, short-read coverage was restricted to haplotype 1 of chromosome 9, whereas *Ren6*^−^ accessions showed coverage only on haplotype 2 (Supplementary Figs 1 to 3). Similarly, for *Ren7*^+^ lines, coverage was confined to haplotype 1 of chromosome 19, while *Ren7*^−^ lines mapped to haplotype 2 (Supplementary Figs 2 to 4). These results indicate that *Ren6* and *Ren7* are located on haplotype 1 of chromosomes 9 and 19, respectively, and their PMS counterparts, i.e. PMS alternative haplotypes, reside on haplotype 2. They also indicate that no haplotype switching occurred during the assembly of DVIT2027. In F2-35, coverage profiles were consistent with the inheritance of PMS haplotypes in the F1 siblines, with no evidence of recombination or haplotype switching (Supplementary Figs 5 to 8).

**Fig. 1.**
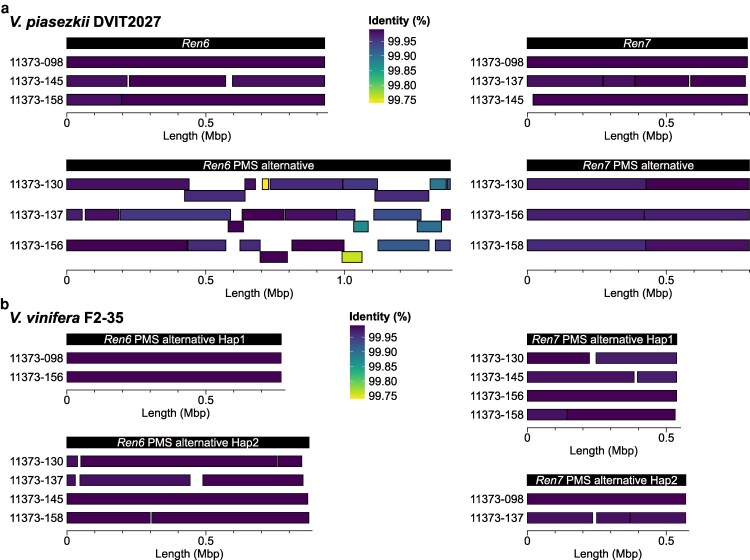
Validation of the haplotype phasing and structure of *Ren6* and *Ren*7 loci and their PM-susceptible (PMS) alternative haplotypes using progeny genomes. Alignment of the genome assemblies of 6 11373 siblines onto *Ren6*, *Ren7*, and their PMS alternatives from *V. piasezkii* DVIT2027 (a) and *V. vinifera* F2-35 (b). Each square represents a contig sequence.

To further validate the phasing, structure, and sequence of the *Ren6* and *Ren7* loci and their PMS alternative haplotypes, we sequenced the genomes of 6 of the 8 siblines using PacBio CLR reads. Alignment of the long DNA-seq reads onto the genomes of DVIT2027 and F2-35 confirmed correct haplotype phasing. Consistent and even read coverage across these regions indicated no misassemblies that could compromise sequence structure (Supplementary Figs 9 and 10). We then assembled the 6 sibline genomes using a trio-binning approach (Koren et al. 2018), generating 2 haploid, parental-phased assemblies per line: one inherited from DVIT2027 and the other from F2-35 (Supplementary Table 4). Alignment of these assemblies to the *Ren6*, *Ren7*, and PMS haplotypes showed complete coverage of the corresponding loci in all 6 siblines (Fig. 1). The only exception was the *Ren6*-PMS haplotype of DVIT2027, which was not fully spanned by a single contig in one sibline. Overall, the alignments showed high sequence similarity, with an average identity of 99.96 ± 0.04% (Fig. 1).

Together, these results validate the phasing and structure of the haplotypes in both parents, *V. piasezkii* DVIT2027 and *V. vinifera* F2-35. In the DVIT2027 genome, the *Ren6* and *Ren7* loci measured 927.5 and 791.6 kbp, respectively, while their PMS alternative haplotypes were 1,380.1 and 771.1 kbp (Table 2). In F2-35, the PMS alternative haplotypes corresponding to *Ren6* and *Ren7* were shorter than those of DVIT2027, measuring 771.1 and 870.7 kbp for *Ren6*-PMS haplotypes, and 537.4 and 570.3 kbp for *Ren7*-PMS haplotypes (Fig. 1).

**Table 2.** Characteristics of the *Ren6* and *Ren7* loci and their alternative PM-susceptible (PMS) haplotypes in *V. piasezkii* DVIT2027 and *V. vinifera* F2-35.

Grape accession	*V. piasezkii* DVIT2027				*V. vinifera* F2-35			
Locus	*Ren6*	*Ren6*-PMS alternative	*Ren7*	*Ren7-*PMS alternative	*Ren6-*PMS alternative Hap1	*Ren6-*PMS alternative Hap2	*Ren7-*PMS alternative Hap1	*Ren7-*PMS alternative Hap2
Chromosome	Chr09 Hap1	Chr09 Hap2	Chr19 Hap1	Chr19 Hap2	Chr09 Hap1	Chr09 Hap2	Chr19 Hap1	Chr19 Hap2
Start–stop (bp)	8,626,384 to 9,553,923	9,061,939 to 10,442,008	883,779 to 1,675,391	951,279 to 1,750,630	8,690,011 to 9,461,149	8,080,301 to 8,951,049	904,827 to 1,442,232	843,589 to 1,413,925
Length (bp)	927,540	1,380,070	791,613	799,352	771,139	870,749	537,406	570,337
Protein-coding gene loci	54	80	81	77	42	50	49	56
Total NLR genes	19	35	13	8	12	12	2	3
CC-NBS-LRR	15	25	10	6	10	8	2	2
CC-NBS	2	5	0	1	1	2	0	0
NBS-LRR	2	4	3	1	1	2	0	1
NBS	0	1	0	0	0	0	0	0

### Identification of the NLR genes composing *Ren6*, *Ren7*, and their PMS alternative haplotypes

**Fig. 2.**
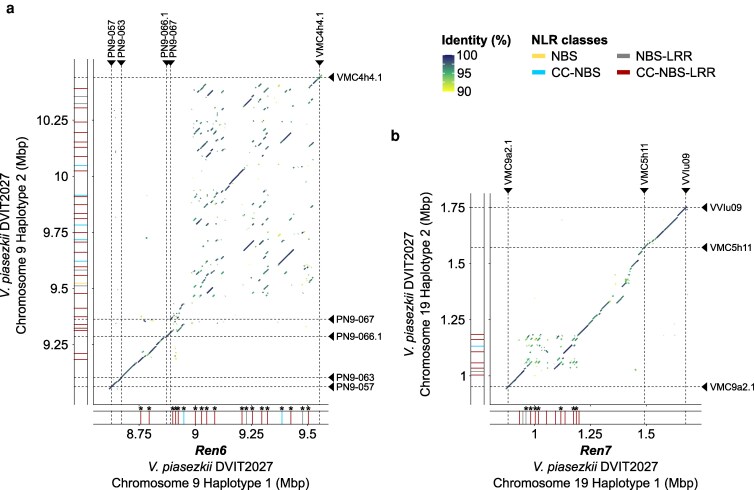
Effect of the structural variations between *Ren6* and *Ren7* loci and their PM-susceptible (PMS) alternative haplotypes on the NLR gene content. Alignment of the PMS alternative haplotypes of *Ren6* (a) and *Ren7* (b) from *V. piasezkii* DVIT2027 onto their PM-resistant counterparts. Chromosomal positions of the NLR genes composing the 4 haplotypes are represented by colored rectangles, whose color indicates the NLR class. NLR genes in *Ren6* and *Ren7* whose protein-coding sequence is impacted by a structural variation are indicated by an asterisk. Panels a and b share the same legend.

Gene annotation, combined with NLR gene prediction and manual refinement of gene models, revealed differences in gene content, particularly NLR genes, between the *R* loci and their PMS counterparts (Table 2). Nineteen and 13 NLR genes were annotated within the *Ren6* and *Ren7* loci, respectively. In *Ren6*, 2 NLR genes were located between markers PN9-063 and PN9-066.1 (202.6 kbp), while the remaining NLR genes clustered between PN9-067 and VMC4h4.1 (664.3 kbp) (Fig. 2a). *Ren6* was mapped between PN9-063 and PN9-067.2 based on QTL analysis (Pap et al. 2016); however, PN9-067.2 and PN9-068 amplified at multiple locations, complicating locus delimitation and reducing the resolution for identifying candidate *R* genes (Supplementary Table 13). Notably, the *Ren6*-PMS haplotype of DVIT2027 contained more NLR genes than the PMR haplotype (35 *vs* 20), with the NLR-containing region spanning 1,209.3 kbp compared to 747.9 kbp in the PMR haplotype, accounting for the observed length difference. In contrast, *Ren7* contained more NLR genes in the PMR haplotype than in the PMS counterpart (13 *vs* 8). These NLRs were clustered within 266.2 and 183.9 kbp regions, respectively, located between markers VMC9a2.1 and VMC5h11 (locus spans of 606.5 and 621.4 kbp, respectively) (Fig. 2b), consistent with the previously defined QTL boundaries (Pap et al. 2016). In both *Ren6* and *Ren7*, most NLR genes encoded CC-NBS-LRR proteins (Table 2; Supplementary Table 14). In F2-35, 12 NLR genes were annotated in each *Ren6*-PMS haplotype, while only 2 and 3 NLR genes were identified in the *Ren7*-PMS haplotypes.

### Comparison of *Ren6* and *Ren7* loci with their PMS alternative haplotypes

To identify NLRs potentially associated with *Ren6* and *Ren7* PM resistance, we compared the PMR haplotypes with their respective PMS counterparts. First, we identified structural variations (SVs; > 50 bp). In *Ren6*, SVs between markers PN9-063 and PN9-066.1 were relatively small (1.0 ± 1.6 kbp) with the region spanning 202.6 kbp in the PMR haplotype and 183 kbp in the PMS haplotype (Fig. 2a). In contrast, numerous large SVs (>10 kbp), particularly duplications, were detected between PN9-067 and VMC4h4.1. This region spanned 664.3 kbp in the PMR haplotype and 1,078.9 kbp in the PMS haplotype, largely accounting for the observed length difference. Manual inspection of the SVs affecting the coding sequences (CDSs) of the NLR genes of *Ren6* revealed that all of them were entirely and/or partially duplicated in DVIT2027 PMS alternative haplotype. By annotating the NLR domains in the genome, we found that these duplication events altered the domain composition of 13 NLRs (Fig. 2a; Supplementary Table 15).

For *Ren7*, a large complex SV spanning 133.6 kbp (from 955.0 to 1,088.6 kbp) was detected on haplotype 1 of chromosome 19 in DVIT2027, relative to haplotype 2 (Fig. 2b). This SV disrupted the CDS of 4 NLR genes (Supplementary Table 16), mainly accounting for the difference in NLR gene number between the 2 haplotypes. Duplication events were identified for 7 NLR genes affecting the domain composition of 6 of them (Supplementary Table 16). Comparable SVs were also identified when comparing the PMS haplotypes of F2-35 with the *Ren6* and *Ren7* loci (Supplementary Fig. 11).

In addition to SVs, we identified small polymorphisms, including 46,178 and 20,669 SNPs and 6,629 and 3,928 INDELs (< 50 bp) in *Ren6* and *Ren7*, respectively (Supplementary Tables 17 and 18). Comparison with the CDS of the NLR genes showed that all the NLRs in *Ren6* and *Ren7* were affected by small polymorphisms. Prediction of the effect of the SNPs showed that every NLRs from *Ren6* and *Ren7*, except g332400 from *Ren7* in which only an INDEL was detected, were affected by at least one nonsynonymous SNP (from 7 to 1,079 in *Ren6*, and from 1 to 376 in *Ren7*) (Supplementary Tables 19 and 20). Among the nonsynonymous SNPs, 77 and 33 ones were found to lead to a premature stop codon in 14 and 7 NLRs of *Ren6* and *Ren7*, respectively. These results suggest that the 2 loci are highly polymorphic.

**Fig. 3.**
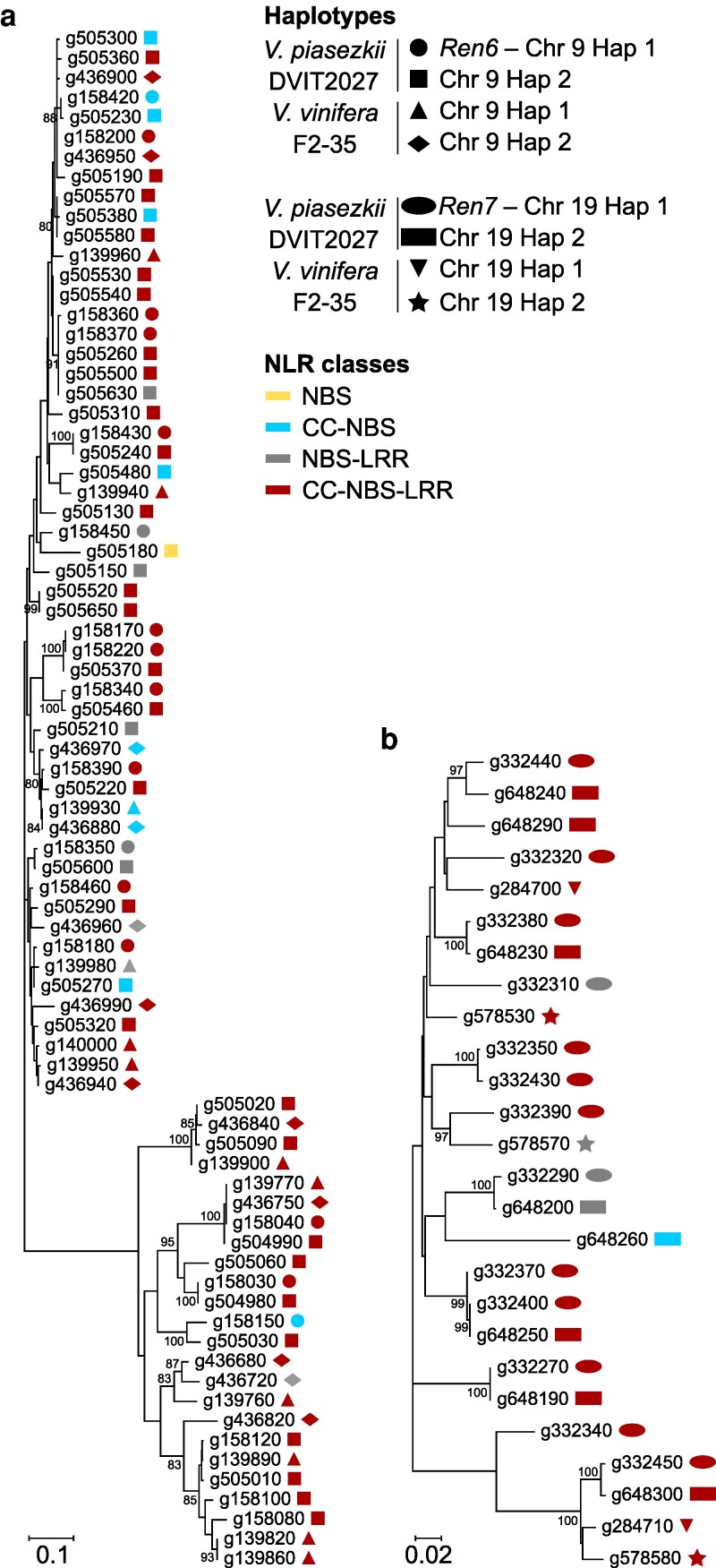
Identification of allele relationships between NLRs composing *Ren6* and *Ren7*, and their PM-susceptible (PMS) alternative haplotypes, based on phylogeny. Neighbor-joining clustering of the predicted NLR sequences composing *Ren6* (a) and *Ren7* (b), and their PMS alternative haplotypes. Scale bar is in the unit of the number of substitutions per site. Bootstrap values greater than 80 are indicated. Panels a and b share the same legend.

To further assess the impact of SVs and small polymorphisms on NLR gene content, we analyzed allelic relationships by constructing phylogenetic trees for the NLR genes at each *R* locus (Fig. 3). In DVIT2027, 2 of the 19 NLR genes (10.5%) in *Ren6* (g158080 and g158100) and 6 of the 13 NLR genes (46.1%) in *Ren7* (g332310, g332320, g332340, g332350, g332390, g332430) were hemizygous, i.e. they lacked allelic counterparts in their respective PMS haplotypes (Supplementary Tables 15 and 16). While some of these genes have allelic counterparts in F2-35, their absence in the PMS haplotypes of DVIT2027 suggests that they may contribute to *Ren6*- and *Ren7*-mediated PM resistance. Noteworthily, no allele pairs shared identical protein sequences, indicating that all NLR genes within *Ren6* and *Ren7* are unique to the PMR haplotypes and distinct from their PMS counterparts in DVIT2027 and F2-35.

Together, these results demonstrate that SVs and small polymorphisms affect both the number and sequence of NLR genes at *Ren6* and *Ren7*, supporting the candidacy of all NLRs in these loci as potential contributors to PM resistance.

### Identification of expressed NLR genes within *Ren6* and *Ren7*

**Fig. 4.**
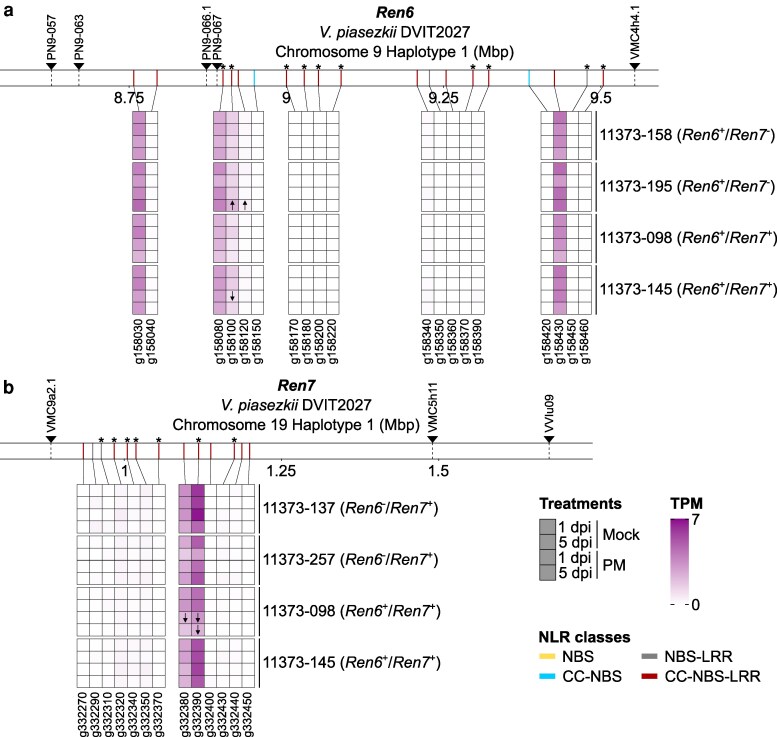
Identification of candidate NLRs responsible for PM resistance within *Ren6* and *Ren7* based on constitutive gene expression. Transcript abundance of the NLR genes belonging to *Ren6* (a) and *Ren7* (b) loci. Gene expression was measured in 11373 siblines possessing either *Ren6, Ren7*, or both *R* loci, at 1 and 5 days postinoculation (dpi) with a mock solution (Mock) or *E. necator* conidia (PM). For each condition, transcript abundance is represented as the mean of transcript per million (TPM) of 3 bioreplicates (*n* = 3). NLR genes more highly and lowly expressed in response to PM are indicated by ↑ and ↓, respectively. Chromosomal positions of the NLR genes composing the 4 haplotypes are represented by colored rectangles, with the color indicating the NLR class. NLR genes whose protein-coding sequence is impacted by at least one structural variation relative to the alternative PMS haplotype of *V. piasezkii* DVIT2027 are indicated by an asterisk. Panels a and b share the same legend.

To narrow down the NLR genes potentially responsible for *Ren6*- and *Ren7*-mediated PM resistance, we analyzed their expression in leaves of 6 11373 siblines carrying *Ren6*, *Ren7*, or both *R* loci. Leaves were sampled 1 and 5 days after inoculation with *E. necator* or a mock solution. Within *Ren6*, 4 CC-NBS-LRR genes were consistently expressed (> 0 TPM) in all *Ren6*^+^ accessions, both constitutively and in response to *E. necator*. One gene (g158030) was located between markers PN9-063 and PN9-066.1, while the other 3 (g158080; g158100; g158430) were located between PN9-067 and VMC4h4.1 (Fig. 4a). Interestingly, 2 of these genes (g158080 and g158100) lack allelic counterparts in the PMS haplotype of DVIT2027. For *Ren7*, 4 CC-NBS-LRR genes were consistently expressed (> 0 TPM) in all the *Ren7*^+^ accessions. Two of them, g332320 and g332350, were expressed at a very low level (0.3 ± 0.1 and 0.2 ± 0.1 TPM, respectively); both lacked allelic counterpart in the PMS haplotype. In contrast, the 2 other CC-NBS-LRR genes, g332380 and g332390, showed higher expression levels (2.7 ± 0.1 and 4.8 ± 1.6 TPM, respectively), with g332390 also missing an allelic counterpart in the PMS haplotype.

To determine the transcriptional modulation of NLRs in *Ren6* and *Ren7* in response to PM, we performed differential gene expression analysis. No NLR gene was differentially expressed in response to PM (*P* < 0.05) in all *Ren6*^+^ or *Ren7*^+^ accessions (Fig. 4). However, within *Ren6*, expression of 2 CC-NBS-LRR genes (g158100 and g158120) was significantly induced (*P* < 0.05) in response to PM in 11373-195 (*Ren6*^+^/*Ren7*^−^) and 11373-145 (*Ren6*^+^/*Ren7*^+^) (Fig. 4a). In *Ren7*, 2 CC-NBS-LRR genes (g332380 and g332390) were significantly downregulated (*P* < 0.05) in 11373-098 (*Ren6*^+^/*Ren7*^+^) following *E. necator* inoculation (Fig. 4b).

**Fig. 5.**
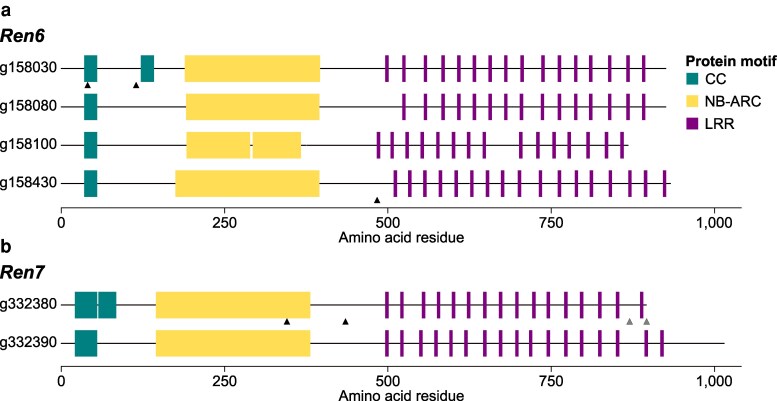
Protein motif composition of the candidate CC-NBS-LRRs associated with the PM resistance of *Ren6* (a) and *Ren7* (b) of *V. piasezkii* DVIT2027. Black and gray triangles indicate the position of the amino changes and deletions compared to the allelic counterpart located in the PM-susceptible haplotype of *V. piasezkii* DVIT2027, respectively. Panels a and b share the same legend.

Altogether, our results identify 4 (g158030; g158080; g158100; g158430) and 2 (g332380 and g332390) candidate CC-NBS-LRR genes potentially associated with *Ren6* and *Ren7* resistance, respectively, based on their consistent expression in PMR siblines, and their absence or sequence divergence in the corresponding PMS haplotype (Fig. 5). Notably, g158080 and g158100 (*Ren6*) and g332390 (*Ren7*) lack allelic counterparts in the PMS haplotype of DVIT2027. The proteins encoded by g158030 and g158430 (*Ren6*) differ from their PMS alleles by 2 and 1 amino acid substitutions, respectively, including one in the CC domain of g158030 (Fig. 5a). For g332380, the predicted protein differs from its PMS allele by 2 amino acid substitutions, including one in the NB-ARC domain, and 2 deletions (5 and 91 amino acids) at the C-terminus (Fig. 5b).

### Comparison of *Ren6* and *Ren7* haplotypes across multiple *V. piasezkii* accessions

**Fig. 6.**
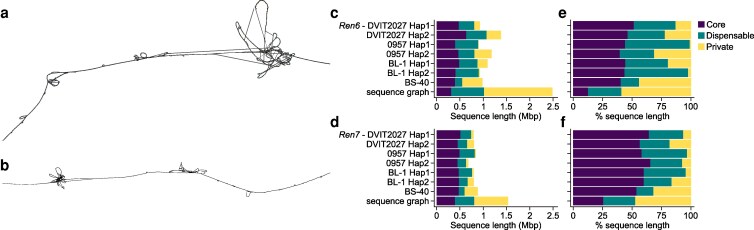
Sequence graphs of *Ren6*, *Ren7*, and their alternative haplotypes across 4 *V. piasezkii* accessions. 2D visualization of the sequence graphs built from *Ren6* (a), *Ren7* (b), and their respective alternative haplotypes in *V. piasezkii* DVIT2027, 0957, BL-1, and BS-40. Cumulative sequence length and percentage of sequence length made of core, dispensable, and private nodes in each *V. piasezkii* haplotype and the sequence graph of the genomic regions corresponding to *Ren6* (c, e) and *Ren7* (d, f). Panels c–f share the same legend.

We compared the *Ren6* and *Ren7* in *V. piasezkii* DVIT2027 with their corresponding regions in 3 additional accessions: 0957 and BL-1, for which haplotype-resolved genomes are available (Guo et al. 2025b), and BS-40, represented by a haploid genome assembly (Zhao et al. 2025). We extracted the genomic regions spanning *Ren6* (PN9-057 to VMC4h4.1) and *Ren7* (VMC9a2.1 to VVIu09) from the 5 haplotypes of accessions 0957, BL-1, and BS-40. The length of the *Ren6*-like haplotypes ranged from 903.6 to 1,245.6 kbp, and *Ren7*-like haplotypes from 681.0 to 881.6 kbp (Fig. 6c, d; Supplementary Tables 21 and 22), indicating structural and sequence variation across accessions.

To characterize this variation in greater detail, we constructed a sequence graph for each locus. The *Ren6* graph, including *Ren6* and 6 alternative haplotypes, comprised 107,811 nodes spanning 2,486,338 bp, while the *Ren7* graph totaled 78,411 nodes over 1,535,147 bp. These graphs capture both shared and haplotype-specific sequences and illustrate structural differences as divergent paths or “bubbles.” Prominent bubbles were observed near the end of the *Ren6* graph and the start of the *Ren7* graph (Fig. 6a, b), consistent with large insertions and duplications in these regions.

To quantify this variation, nodes were classified into 3 categories: (i) core nodes, shared across all 7 haplotypes; (ii) dispensable nodes, present in more than one but not all haplotypes; and (iii) private nodes, unique to a single haplotype. In the *Ren6* graph, private nodes accounted for 59% of the total graph length, dispensable nodes for 28.3%, and core nodes for only 12.7% (Fig. 6e). Similar trends were observed in the *Ren7* graph, though the proportion of private nodes was slightly lower (47.5%) and core nodes higher (25.4%) (Fig. 6f). These results indicate substantial sequence divergence among *V. piasezkii* accessions at both loci. The cumulative length of private nodes varied widely across haplotypes. In the *Ren6* graph, it ranged from 9.0 kbp in 0957 haplotype 1 to 433.3 kbp in the BS-40 haplotype (Fig. 6c). A similar pattern was observed for *Ren7*, with private node lengths ranging from 29.9 kbp in 0957 haplotype 1 to 281.8 kbp in BS-40 (Fig. 6d).

Regarding the candidate NLR genes of DVIT2027, we identified private CDS variants specific to the *Ren6* haplotype in its 4 candidate *R* genes: 2 bp in g158030, 27 bp in g158080, 7 bp in g158100, and 1 bp in g158430. For the *Ren7* candidates, 21 and 35 bp were found to be specific to the *R* locus in g332320 and g332350, respectively. Comparative analysis further revealed that only one NLR gene within *Ren6* (g158460) encoded a protein identical to that found in haplotype 1 of the *V. piasezkii* 0957 genome, underscoring the unique NLR composition of *Ren6* and *Ren7* in DVIT2027.

## Discussion

To better understand the genetic basis of PM resistance in wild grape species, we focused on resolving the structure, sequence diversity, and candidate genes of *Ren6* and *Ren7* in *V. piasezkii* DVIT2027. Because both loci are heterozygous in DVIT2027, it was necessary to distinguish each *R* locus from its alternative, PMS haplotype. *In silico* amplification of *Ren6*- and *Ren7*-associated markers revealed length polymorphisms (Supplementary Tables 21 and 22) that helped differentiate the haplotypes, although the observed lengths differed slightly from the ones previously reported (Pap et al. 2016). However, marker-based haplotype identification alone cannot confirm locus structure or phasing accuracy. To address this, we integrated genomic data from F1 siblines carrying different combinations of *Ren6* and *Ren7,* enabling us to verify haplotype phasing and locus structure. This required aligning sequencing reads onto both parental genomes, prompting us to also generate the diploid genome of the PMS parent of the 11373 population, F2-35. Further support to the structure of the 2 loci came from aligning parental-phased assemblies of 6 progeny back to the parental haplotypes. While *Ren6* and *Ren7*, and their PMS counterparts were fully resolved within single contigs in *V. vinifera* F2-35 and in at least one sibline, the PMS haplotype of *Ren6* in DVIT2027 remained fragmented in all cases (Fig. 1). These results highlight the value of phased diploid assemblies and integrated pipelines that combine long-read sequencing and high-quality phasing, while also underscoring the continued need for manual validation and careful curation of the locus structure and gene models (Cantu et al. 2024). The fragmentation observed in CLR-based assemblies reinforces the shift toward HiFi technology or other high-fidelity technologies for resolving complex *R* loci. Although the alignment of CLR reads and parental-phased genomes of the 6 11373 siblines onto *Ren6*, *Ren7*, and their PMS haplotypes did not show any evidence of phasing errors or misassignments affecting the NLR gene content in the regions, undetected recombination or local assembly artifacts remain possible.

Alignments of the PMS haplotypes onto *Ren6* and *Ren7* showed large and complex SVs in the regions containing the NLR genes, as well as several short polymorphisms (Fig. 2), similar to observations at *Run1.2* and *Run2.2* of *M. rotundifolia* Trayshed (Massonnet et al. 2022b). This supports a model in which NLR clusters in grapevine, as in many angiosperms, diversify at both inter- and intraspecific levels in response to pathogen pressure (Contreras et al. 2023). Phylogenetic analyses (Fig. 3) confirmed that SVs and small polymorphisms lead to presence/absence variation, including NLRs from *Ren6* and *Ren7* without allelic counterparts in their PMS haplotypes. All NLR protein sequences in these loci were unique to the PMR haplotypes. The uniqueness of the NLR protein sequences of both *R* loci showed that sequence specificity, especially in the NLR domains, is more crucial in disease resistance rather than the number of NLRs; particularly in the case of *Ren6*, for which the PMS haplotype contains more NLRs than the PMR haplotype. This also reinforces the importance of validating phasing and structure before inferring candidate genes and highlights the limitations of relying on reference genomes from related species or accessions that do not carry the same *R* loci (Paineau et al. 2024).

This point is further supported by our analysis of intraspecific sequence diversity at *Ren6* and *Ren7* using sequence graphs built from 4 *V. piasezkii* accessions. These graphs revealed substantial haplotype variation, including private sequence nodes within candidate NLRs, reinforcing the importance of generating diploid genomes from the actual resistance source. The low proportion of core sequence (12.7% in *Ren6* graph and 25.4% in *Ren7* graph) and high fraction of sequence composed of private nodes (up to 59%) indicate that most of the sequence content at these loci is accession- or haplotype-specific. Such patterns point to ongoing structural remodeling within *V. piasezkii R* loci, likely driven by pathogen selection. Notably, the BS-40 haplotype carried the largest cumulative length of private nodes, highlighting how distinct *R* locus architectures can emerge even within a single species. This divergence extended to the candidate NLR genes: several CDS variants were unique to the DVIT2027 haplotypes, and only one gene (g158460) had an identical protein sequence in another accession (0957). These findings underscore the unique genetic makeup of *Ren6* and *Ren7* in DVIT2027 and caution against relying on reference genomes from non-resistant accessions. Additional analyses of the accessions included in this study, and other PMR *V. piasezkii* accessions such as DVIT2026, DVIT2028, and DVIT2032 (Pap et al. 2016), could help determine whether they share PMR haplotypes and uncover additional resistance alleles or genes.

The structural variation and gene presence/absence patterns at *Ren6* and *Ren7* are likely driven by localized duplications, deletions, and transposable element (TE) activity. These mechanisms, along with unequal crossing-over, are well-known contributors to the dynamic architecture of NLR clusters (Jacob et al. 2013; Contreras et al. 2023). Similar patterns have been reported at *Run1* (Feechan et al. 2013), *Run1.2* and *Run2.2* (Cochetel et al. 2021; Massonnet et al. 2022b), *Rpv3* (Wilkerson et al. 2025), and *Rpv33* (Zou et al. 2023), suggesting that dynamic NLR evolution is a shared feature of grape *R* loci. With the increasing availability of phased diploid genomes from diverse *Vitis* species (Cantu et al. 2024), we are now positioned to conduct systematic comparisons across the genus. These comparisons will clarify NLR gene birth and loss, the role of TEs and recombination, and the evolutionary pressures shaping resistance loci (Jacob et al. 2013; Contreras et al. 2023; Guo et al. 2025a, 2025b).

In addition to using genomes of 11373 siblines in our pipeline for the identification of candidate NLR genes, we integrated transcriptomic data from leaves of 6 11373 siblines carrying *Ren6*, *Ren7*, or both *R* loci, in the presence or absence of the pathogen. Based on constitutive expression (Fig. 4) and absence or sequence divergence relative to the PMS haplotype (Fig. 5), we identified 4 (g158030; g158080; g158100; g158430) and 2 (g332380 and g332390) candidate CC-NBS-LRR genes associated with *Ren6* and *Ren7* resistance, respectively. These candidates varied in CC domain number (1 or 2) and number of LRR motifs (15 to 17), which may influence effector recognition specificity (McHale et al. 2006). Transcriptomic data from additional recombinant lines and baseline (0 dpi) samples will help confirm and refine these candidates, especially in *Ren6*, where some candidate genes are separated by > 500 kbp.

Future work will focus on functional validation of these candidates using transient or stable transformation methods. Transient tools available in grapevine include *Agrobacterium*-mediated expression (Jiang et al. 2021; Zhang et al. 2024), virus-induced gene silencing (Yang et al. 2022), and exogenous dsRNA-based RNAi (Marcianò et al. 2021). Stable methods such as gene editing, complementation, or targeted knock-ins remain limited but are advancing (Greenwood et al. 2023; Schreiber et al. 2024). Validating candidate NLRs and testing their response to a broader panel of *E. necator* isolates will enable the development of perfect markers for breeding and guide editing strategies to stack or fine-tune resistance traits while preserving varietal identity (Ren et al. 2024).

From a breeding perspective, the sequence-level resolution of *Ren6* and *Ren7* and their candidate genes provides a foundation for precise marker development. Given the complete haplotype specificity of these NLRs, such markers could enable rapid and accurate screening in breeding programs or guide-targeted gene editing efforts (Greenwood et al. 2023). The future developed markers will need to be tested in independent populations segregating for *Ren6* and *Ren7* to validate their specificity and association with PM resistance. Stacking *Ren6* and *Ren7* with other *R* loci, such as *Run1* and *Ren4*, is already underway in multiple programs to enhance resistance durability and mitigate the risk of resistance breakdown.

## Supplementary Material

jkaf250_Supplementary_Data

## Data Availability

Sequencing data are accessible through NCBI under the BioProject PRJNA1258375. Genome sequences and their annotation files are available at NCBI under the BioProjects PRJNA1337268-PRJNA1337271 and at Zenodo doi:10.5281/zenodo.15361017. Supplemental material available at G3 online.
