# Supplementary material for: Dissection of the Ren6 and Ren7 powdery mildew resistance loci in Vitis piasezkii DVIT2027 using phased parental–progeny genomes and intraspecific locus graph reconstruction
Source: G3 (Bethesda). 2025 Oct 21;15(12):jkaf250. doi: 10.1093/g3journal/jkaf250 (PMC12693563; doi:10.1093/g3journal/jkaf250)
Supplement: jkaf250_Supplementary_Data [file jkaf250_supplementary_data.zip › Supplemental_Figures_G3-2025-406273.pdf]

Dissection of the *Ren6* and *Ren7* powdery mildew resistance loci in *Vitis piasezkii* DVIT2027 using phased parental–progeny genomes and intraspecific locus graph reconstruction

Mélanie Massonnet[1], Rosa Figueroa-Balderas[1], Noé Cochetel[1], Summaira Riaz[1], Dániel Pap[1], M. Andrew Walker[1], Dario Cantu[1,2*]

[1]Department of Viticulture and Enology, University of California Davis, Davis, CA, 956161, USA
[2]Genome Center, University of California Davis, Davis, CA, 95616, USA

[*]Email: dacantu@ucdavis.edu

Supplementary figures

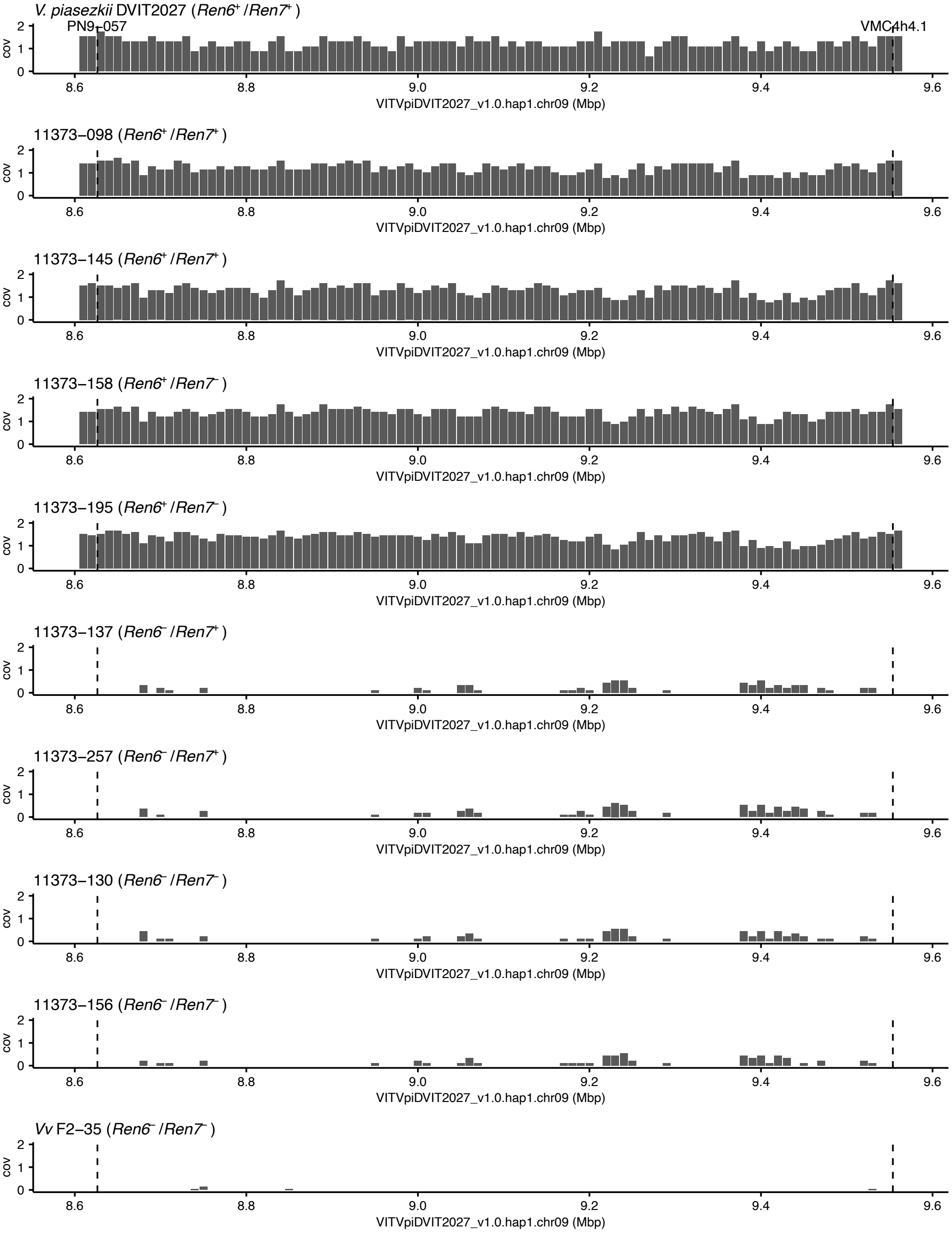

Figure S1: Checking of the haplotype phasing of *Ren6* in *V. piasezkii* DVIT2027 genome using short DNA-seq reads. Normalized median base coverage per 10 kbp of *V. piasezkii* DVIT2027, eight 11373 sib-lines, and *V. vinifera* F2-35 (*Vv* F2-35), at the *Ren6* locus of *V. piasezkii* DVIT2027. Only DNA-seq reads out of repetitive elements and aligning perfectly were used for base coverage analysis.

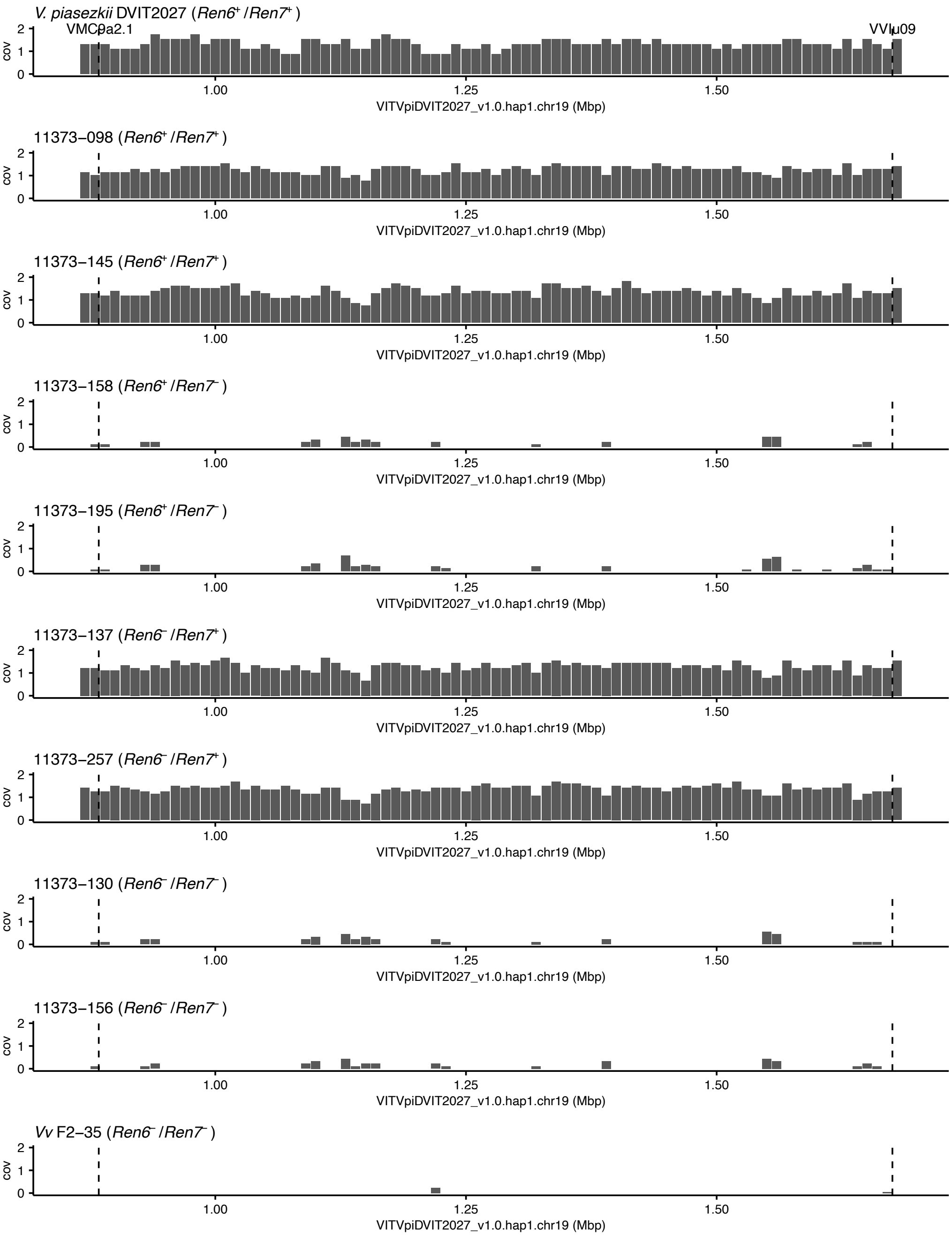

Figure S2: Checking of the haplotype phasing of *Ren7* in *V. piasezkii* DVIT2027 genome using short DNA-seq reads. Normalized median base coverage per 10 kbp of *V. piasezkii* DVIT2027, eight 11373 sib-lines, and *V. vinifera* F2-35 (*Vv* F2-35), at the *Ren7* locus of *V. piasezkii* DVIT2027. Only DNA-seq reads out of repetitive elements and aligning perfectly were used for base coverage analysis.

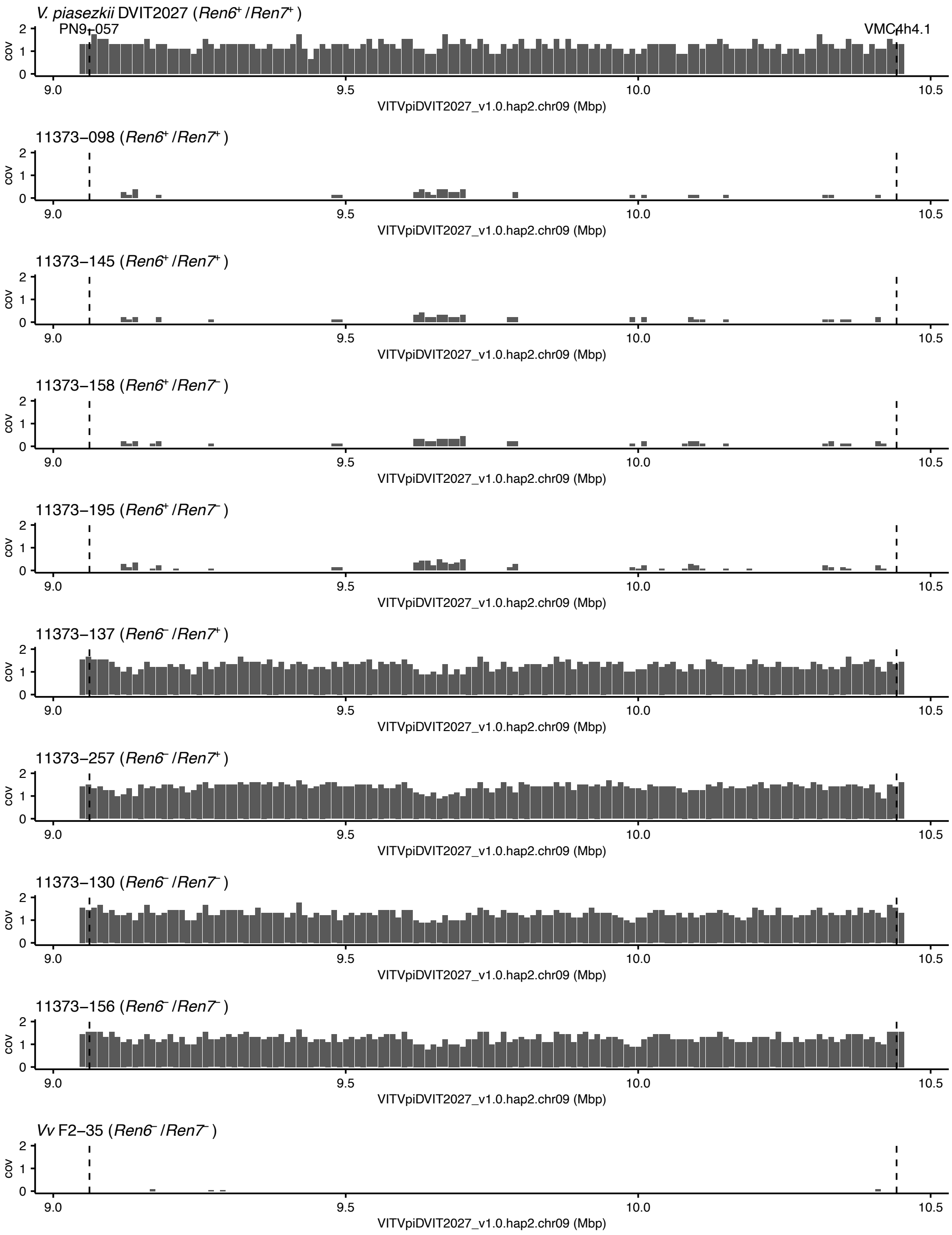

Figure S3: Checking of the haplotype phasing of the PM-susceptible alternative haplotype of *Ren6* in *V. piasezkii* DVIT2027 genome using short DNA-seq reads. Normalized median base coverage per 10 kbp of *V. piasezkii* DVIT2027, eight 11373 sib-lines, and *V. vinifera* F2-35 (*Vv* F2-35), at the PM-susceptible alternative haplotype of *Ren6* locus of *V. piasezkii* DVIT2027. Only DNA-seq reads out of repetitive elements and aligning perfectly were used for base coverage analysis.

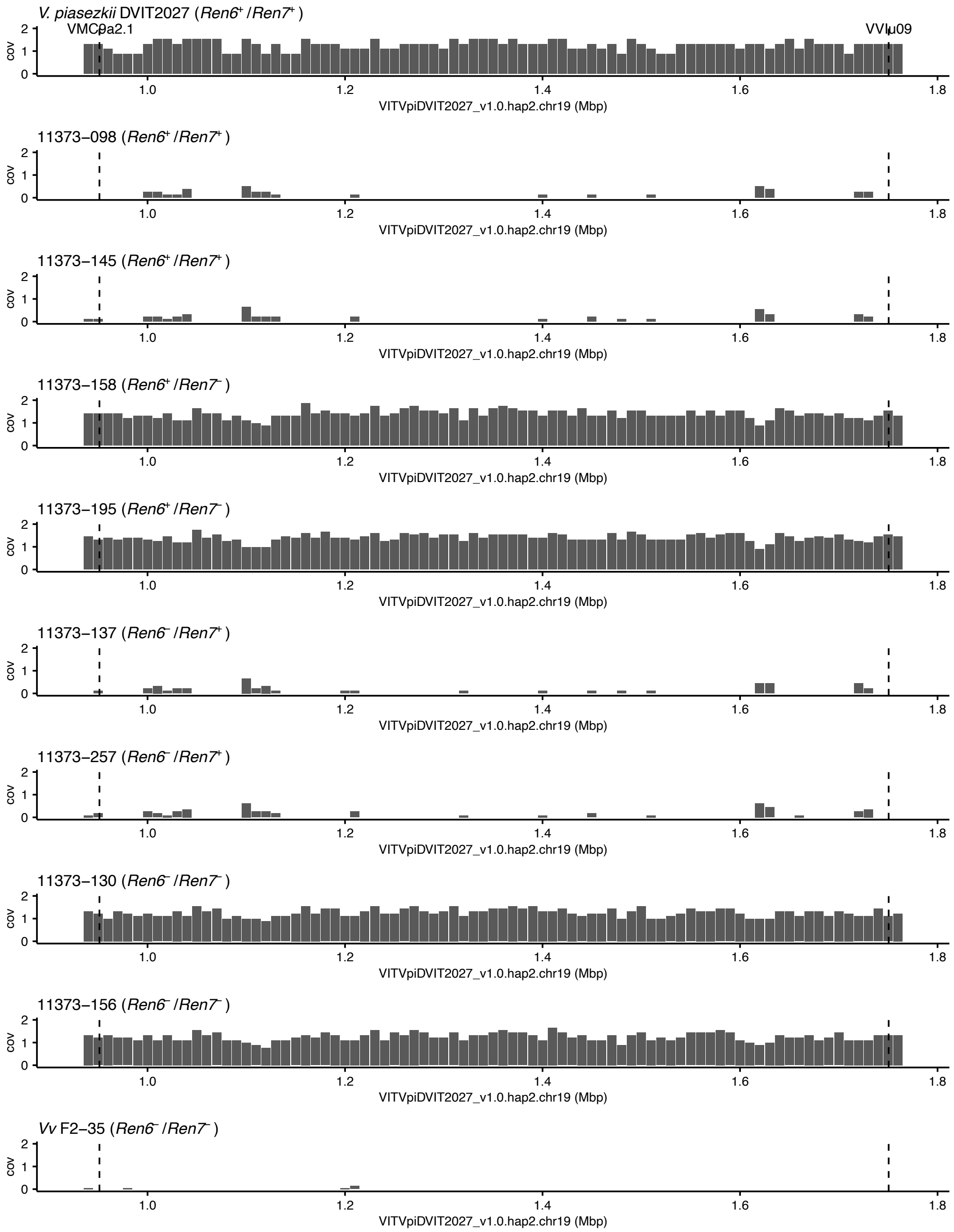

Figure S4: Checking of the haplotype phasing of the PM-susceptible alternative haplotype of *Ren7* in *V. piasezkii* DVIT2027 genome using short DNA-seq reads. Normalized median base coverage per 10 kbp of *V. piasezkii* DVIT2027, eight 11373 sib-lines, and *V. vinifera* F2-35 (*Vv* F2-35), at the PM-susceptible alternative haplotype of *Ren7* locus of *V. piasezkii* DVIT2027. Only DNA-seq reads out of repetitive elements and aligning perfectly were used for base coverage analysis.

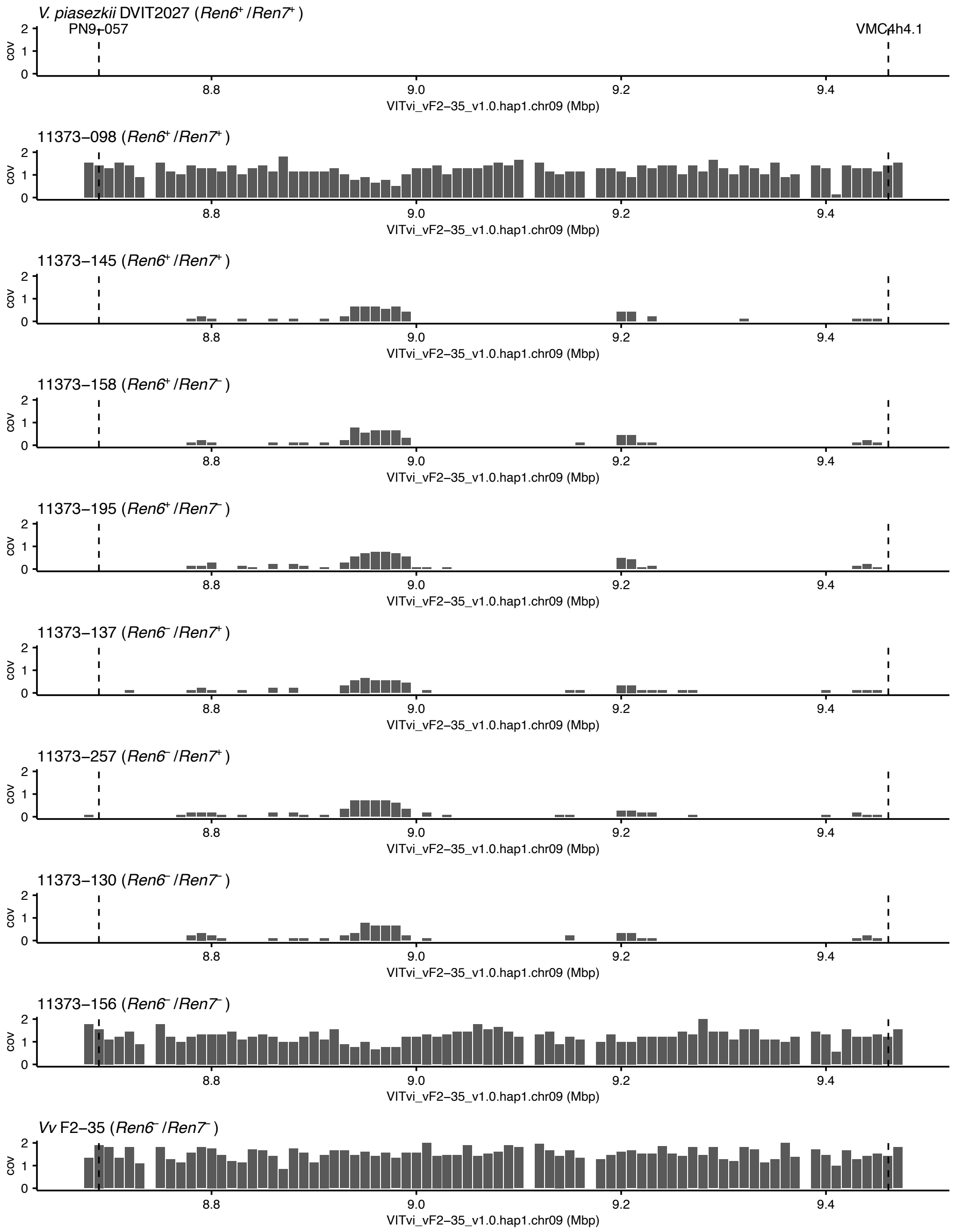

Figure S5: Checking of the haplotype phasing of the PM-susceptible alternative haplotype 1 of *Ren6* in *V. vinifera* F2-35 genome using short DNA-seq reads. Normalized median base coverage per 10 kbp of *V. piasezkii* DVIT2027, eight 11373 sib-lines, and *V. vinifera* F2-35 (*Vv* F2-35), at the PM-susceptible alternative haplotype 1 of *Ren6* locus of *V. vinifera* F2-35. Only DNA-seq reads out of repetitive elements and aligning perfectly were used for base coverage analysis.

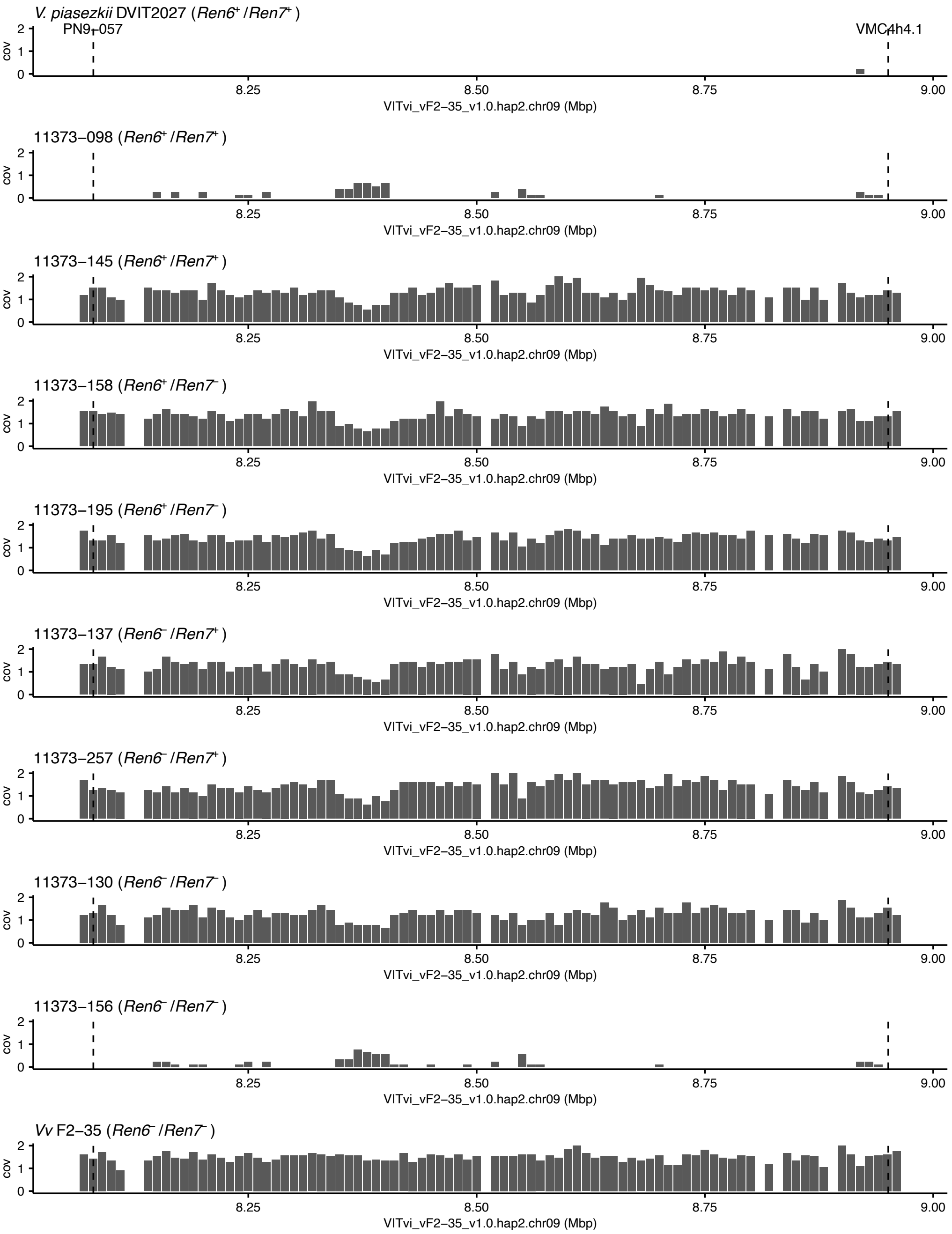

Figure S6: Checking of the haplotype phasing of the PM-susceptible alternative haplotype 2 of *Ren6* in *V. vinifera* F2-35 genome using short DNA-seq reads. Normalized median base coverage per 10 kbp of *V. piasezkii* DVIT2027, eight 11373 sib-lines, and *V. vinifera* F2-35 (*Vv* F2-35), at the PM-susceptible alternative haplotype 2 of *Ren6* locus of *V. vinifera* F2-35. Only DNA-seq reads out of repetitive elements and aligning perfectly were used for base coverage analysis.

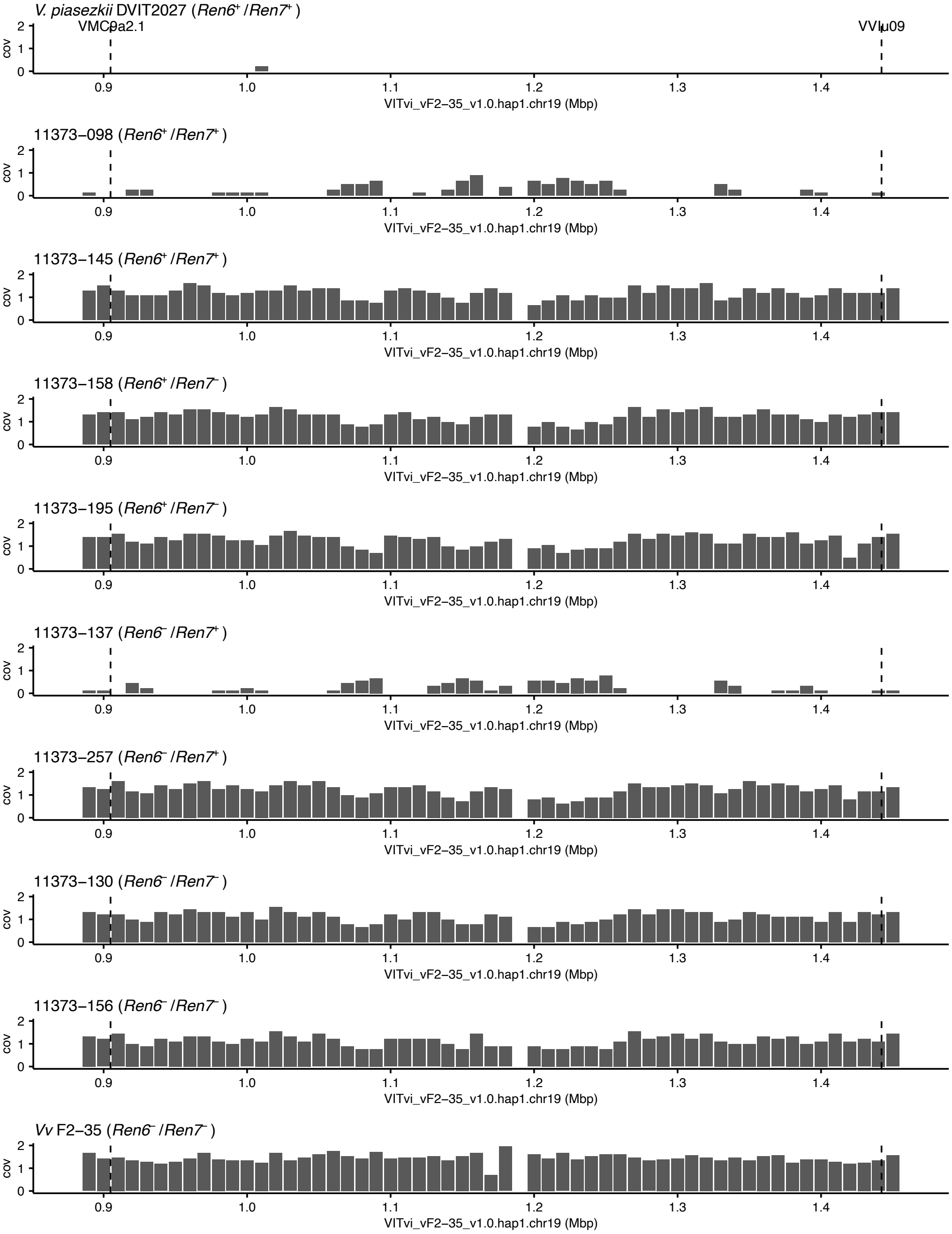

Figure S7: Checking of the haplotype phasing of the PM-susceptible alternative haplotype 1 of *Ren7* in *V. vinifera* F2-35 genome using short DNA-seq reads. Normalized median base coverage per 10 kbp of *V. piasezkii* DVIT2027, eight 11373 sib-lines, and *V. vinifera* F2-35 (*Vv* F2-35), at the PM-susceptible alternative haplotype 1 of *Ren7* locus of *V. vinifera* F2-35. Only DNA-seq reads out of repetitive elements and aligning perfectly were used for base coverage analysis.

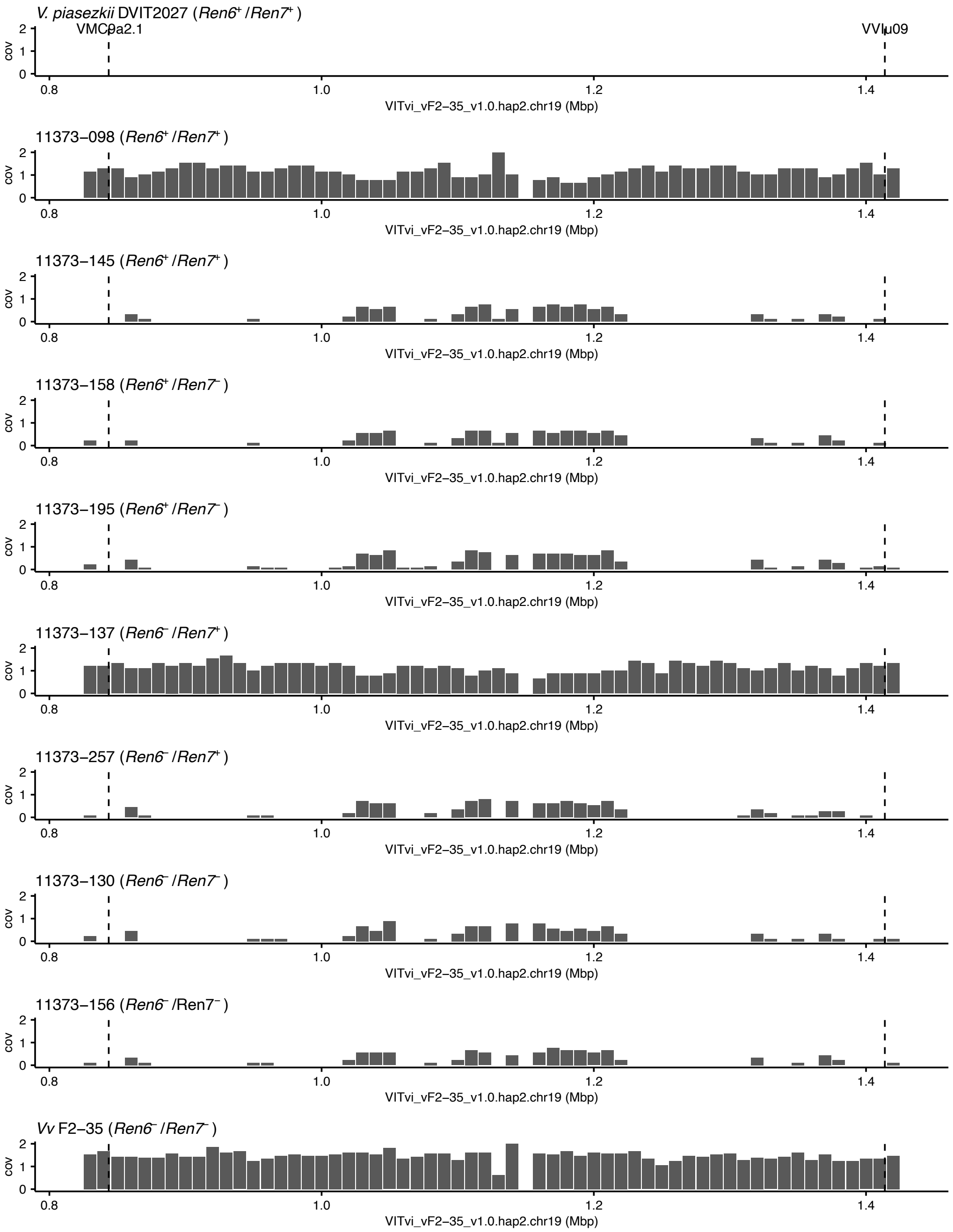

Figure S8: Checking of the haplotype phasing of the PM-susceptible alternative haplotype 2 of *Ren7* in *V. vinifera* F2-35 genome using short DNA-seq reads. Normalized median base coverage per 10 kbp of *V. piasezkii* DVIT2027, eight 11373 sib-lines, and *V. vinifera* F2-35 (*Vv* F2-35), at the PM-susceptible alternative haplotype 2 of *Ren7* locus of *V. vinifera* F2-35. Only DNA-seq reads out of repetitive elements and aligning perfectly were used for base coverage analysis.

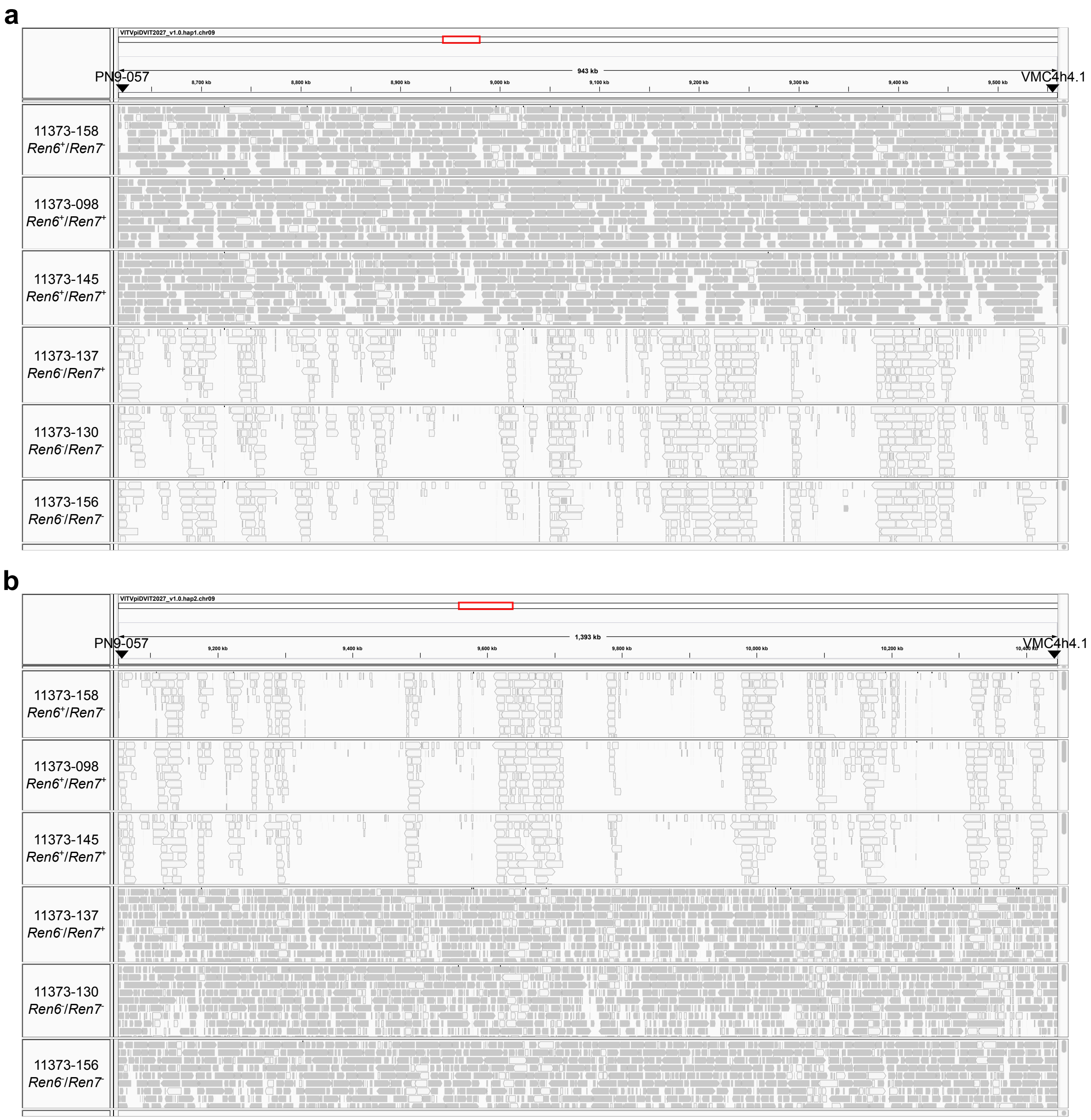

Figure S9: Checking of the haplotype phasing and the structure of *Ren6* and its PMS alternative haplotype in *V. piasezkii* DVIT2027 genome using PacBio CLR reads. Alignment of the long DNA-seq reads from six 11373 sib-lines at *Ren6* (**a**) and its PMS alternative haplotype (**b**) of *V. piasezkii* DVIT2027.

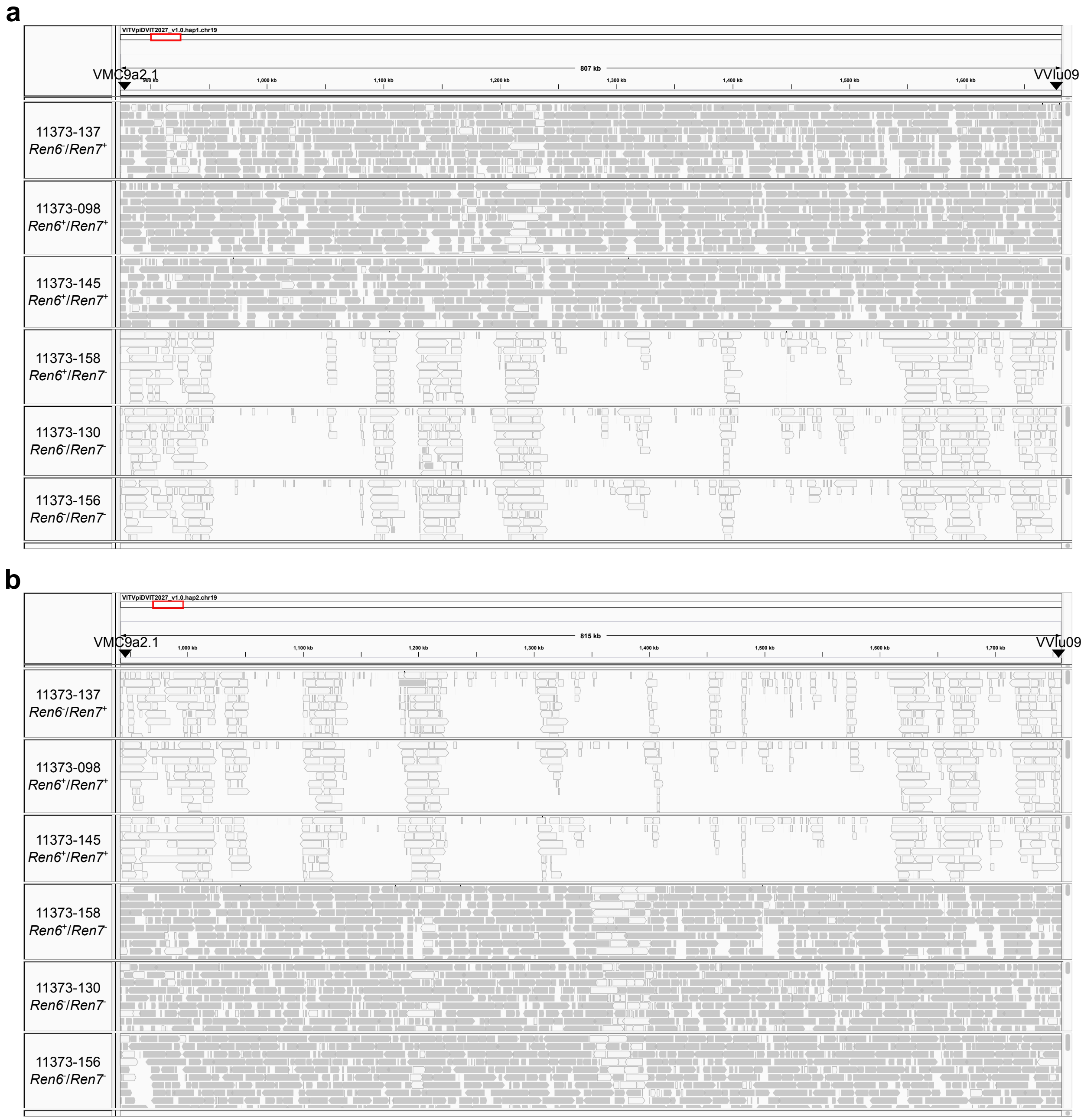

Figure S10: Checking of the haplotype phasing and the structure of *Ren7* and its PMS alternative haplotype in *V. piasezkii* DVIT2027 genome using PacBio CLR reads. Alignment of the long DNA-seq reads from six 11373 sib-lines at *Ren7* (**a**) and its PMS alternative haplotype (**b**) of *V. piasezkii* DVIT2027.

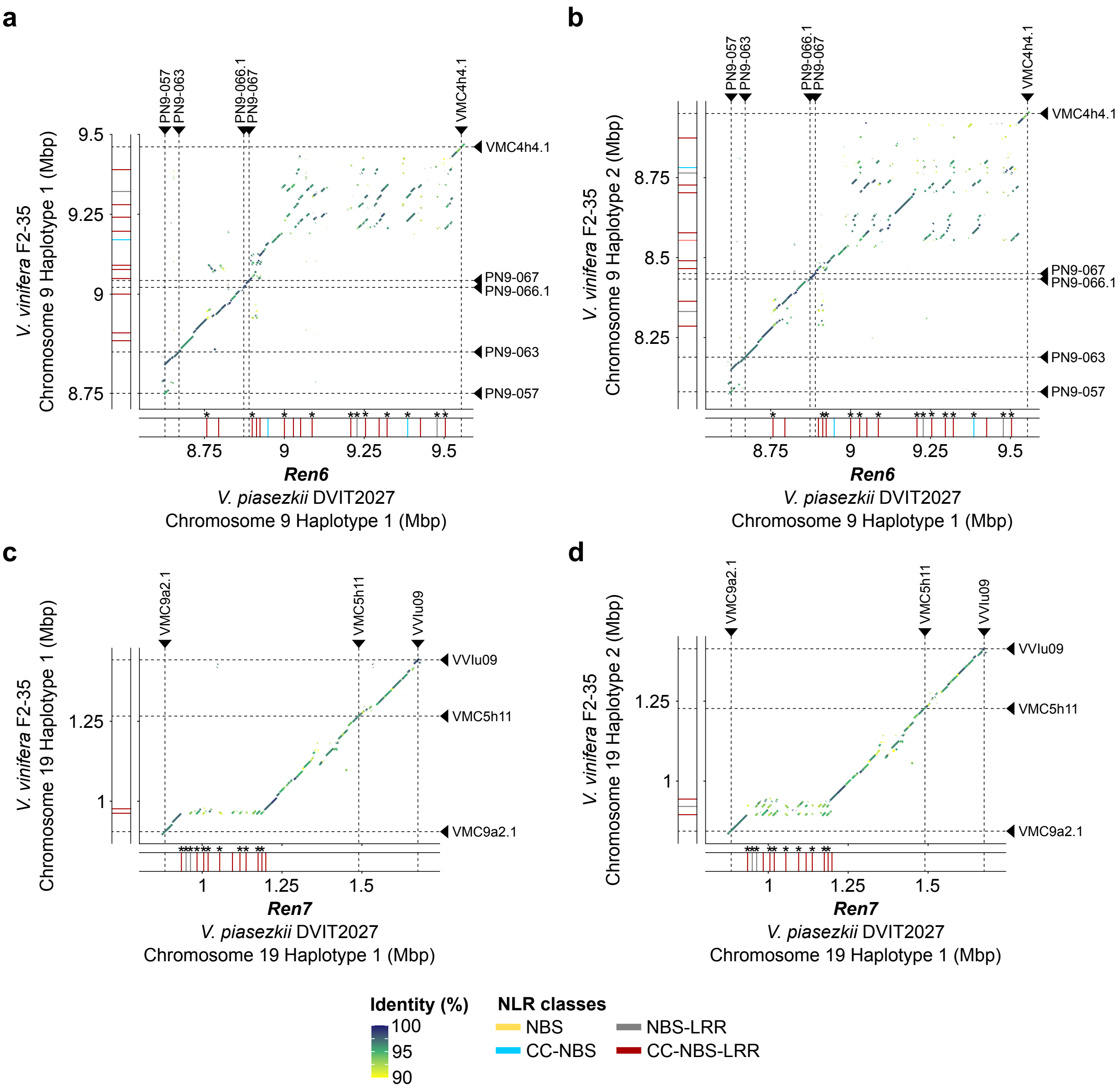

Figure S11: Effect of the structural variations between *Ren6* and *Ren7* loci and their PM-susceptible alternative haplotypes in *V. vinifera* F2-35 on the NLR gene content. Alignment of the PM-susceptible alternative haplotypes of *Ren6* (**a**) and *Ren7* (**b**) from *V. vinifera* F2-35 onto their PM-resistant counterparts from *V. piasezkii* DVIT2027. Chromosomal positions of the NLR genes composing the haplotypes are represented by colored rectangles, which color indicates the NLR class. NLR genes in *Ren6* and *Ren7* which protein-coding sequence is impacted by a structural variation are indicated by an asterisk.
